# Novel roles of PIWI proteins and PIWI-interacting RNAs in human health and diseases

**DOI:** 10.1186/s12964-023-01368-x

**Published:** 2023-11-29

**Authors:** Zeyu Wu, Xiao Yu, Shuijun Zhang, Yuting He, Wenzhi Guo

**Affiliations:** 1 Department of Hepatobiliary and Pancreatic Surgery, The First Affiliated Hospital of Zhengzhou University, Zhengzhou, 450052 China; 2 Key Laboratory of Hepatobiliary and Pancreatic Surgery and Digestive Organ Transplantation of Henan Province, The First Affiliated Hospital of Zhengzhou University, Zhengzhou, 450052 China; 3 Open and Key Laboratory of Hepatobiliary & Pancreatic Surgery and Digestive Organ Transplantation at Henan Universities, Zhengzhou, 450052 China; 4 Henan Key Laboratory of Digestive Organ Transplantation, Zhengzhou, 450052 China

**Keywords:** piRNA, PIWI, Human diseases, Cancer, Functional mechanism

## Abstract

Non-coding RNA has aroused great research interest recently, they play a wide range of biological functions, such as regulating cell cycle, cell proliferation, and intracellular substance metabolism. Piwi-interacting RNAs (piRNAs) are emerging small non-coding RNAs that are 24–31 nucleotides in length. Previous studies on piRNAs were mainly limited to evaluating the binding to the PIWI protein family to play the biological role. However, recent studies have shed more lights on piRNA functions; aberrant piRNAs play unique roles in many human diseases, including diverse lethal cancers. Therefore, understanding the mechanism of piRNAs expression and the specific functional roles of piRNAs in human diseases is crucial for developing its clinical applications. Presently, research on piRNAs mainly focuses on their cancer-specific functions but lacks investigation of their expressions and epigenetic modifications. This review discusses piRNA’s biogenesis and functional roles and the recent progress of functions of piRNA/PIWI protein complexes in human diseases.

Video Abstract

**Supplementary Information:**

The online version contains supplementary material available at 10.1186/s12964-023-01368-x.

## Introduction

Human health achieves dynamic balance through various adjustments. The homeostasis of the internal environment is a necessary condition for normal life activities [1–3]. Disturbances in the internal environment cause to mutations and disruption of the balance of various genes, leading to a variety of human diseases, including cancer and cardiovascular and cerebrovascular diseases that kill millions yearly [4–7]. Normal functional activities of cells support the homeostasis of the internal environment, essentially activated by various intracellular signaling pathways and epigenetic modifications [2, 8, 9].

PIWI-interacting RNAs (piRNAs), the least studied class of ncRNAs to date, is an emerging class of ncRNAs ranging in length from 24 to 32 nucleotides and was first identified in Drosophila, mouse, and rat germ cells in 2006 [9–11]. piRNA, initially identified as highly expressed in a germ-line cell, maintains genomic integrity [12–14]. More in-depth research shows piRNA to be widely expressed in somatic cells and engaged in some fundamental regulations [15, 16], such as cell cycle regulation, proliferation, energy metabolism and immune microenvironment regulation through epigenetic modification and regulation of multiple signaling pathways [17, 18]. The functional roles of piRNA in germ and somatic cells have greatly interested researchers for further studies.

Growing bodies of evidence have revealed the essential functions of piRNA in the occurrence and prognosis of various human diseases and its application prospect in diagnosing and treating related conditions [19–21]. In addition, piRNA/piwi complexes have been shown to bind piRNA to piwi proteins and to perform regulatory functions such as transposon silencing, epigenetic modification, and protein regulation [22, 23]. Moreover, piRNA plays a vital role as a diagnostic marker in the early stage of various cancers, serving as critical prognostic indicators after treatment [24–26]. At the same time, piRNA/piwi proteins enhance the resistance of tumor cells to currently known chemotherapeutics, indicating the possibility of it being a new therapeutic target that can potentially solve the problem of chemotherapy resistance [27].

**Fig. 1:**
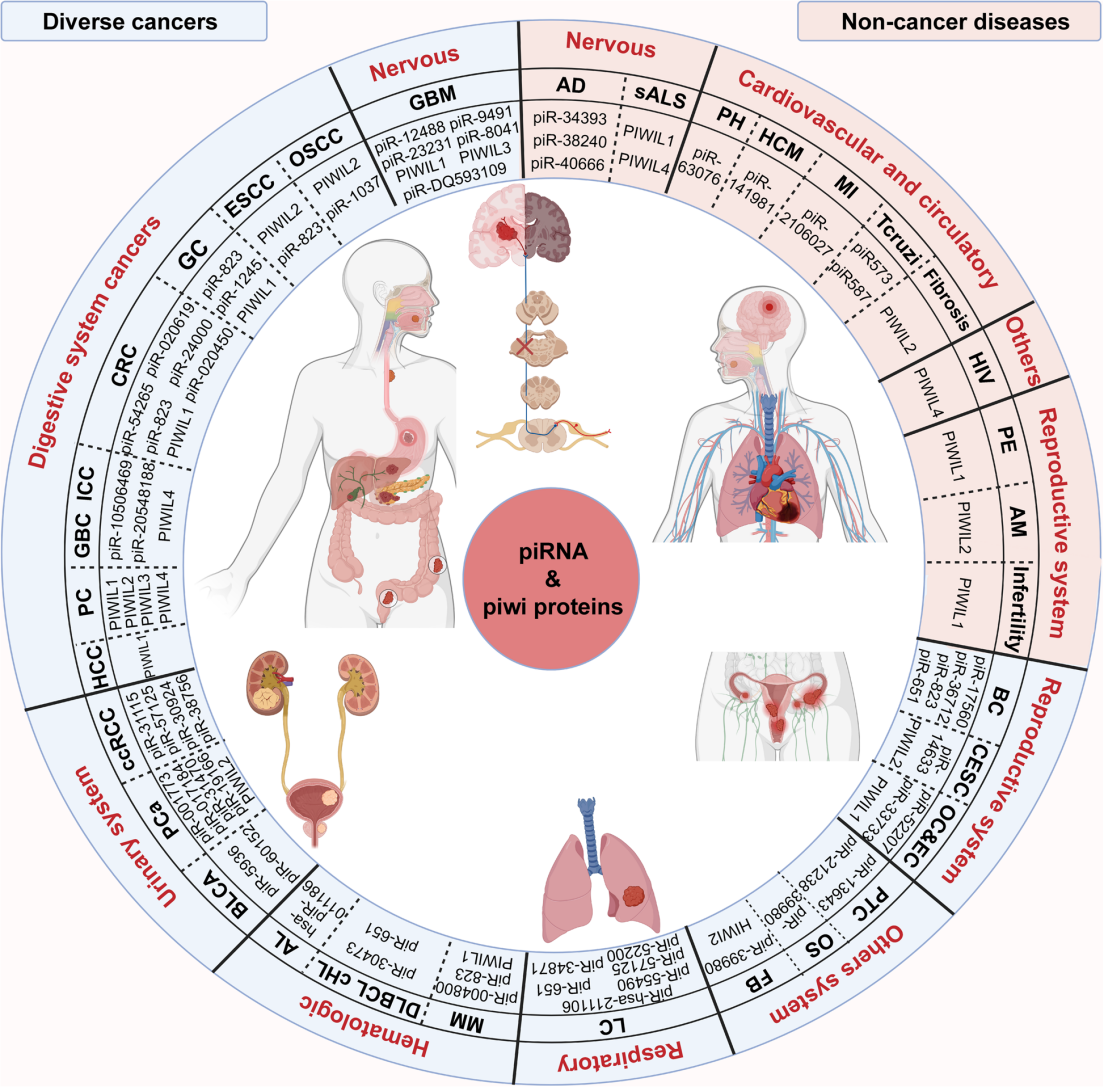
The association of abnormally dysregulated piRNAs/PIWI proteins with human diseases can be broadly divided into two categories. On the one hand, the blue background of piRNA/PIWI proteins represent their important roles in the development of different human cancers. On the other hand, the pink background of piRNA/PIWI proteins represent the correlation in the occurrence and development of human non-cancer diseases

This review summarized the latest progress of piRNA biogenesis research. It expounded on the biological functions and clinical value of piRNA in recent years to provide a systematic summary of the potential role of piRNA in human diseases (Fig. 1).

## Biosynthesis and basic functions of piRNAs

**Fig. 2:**
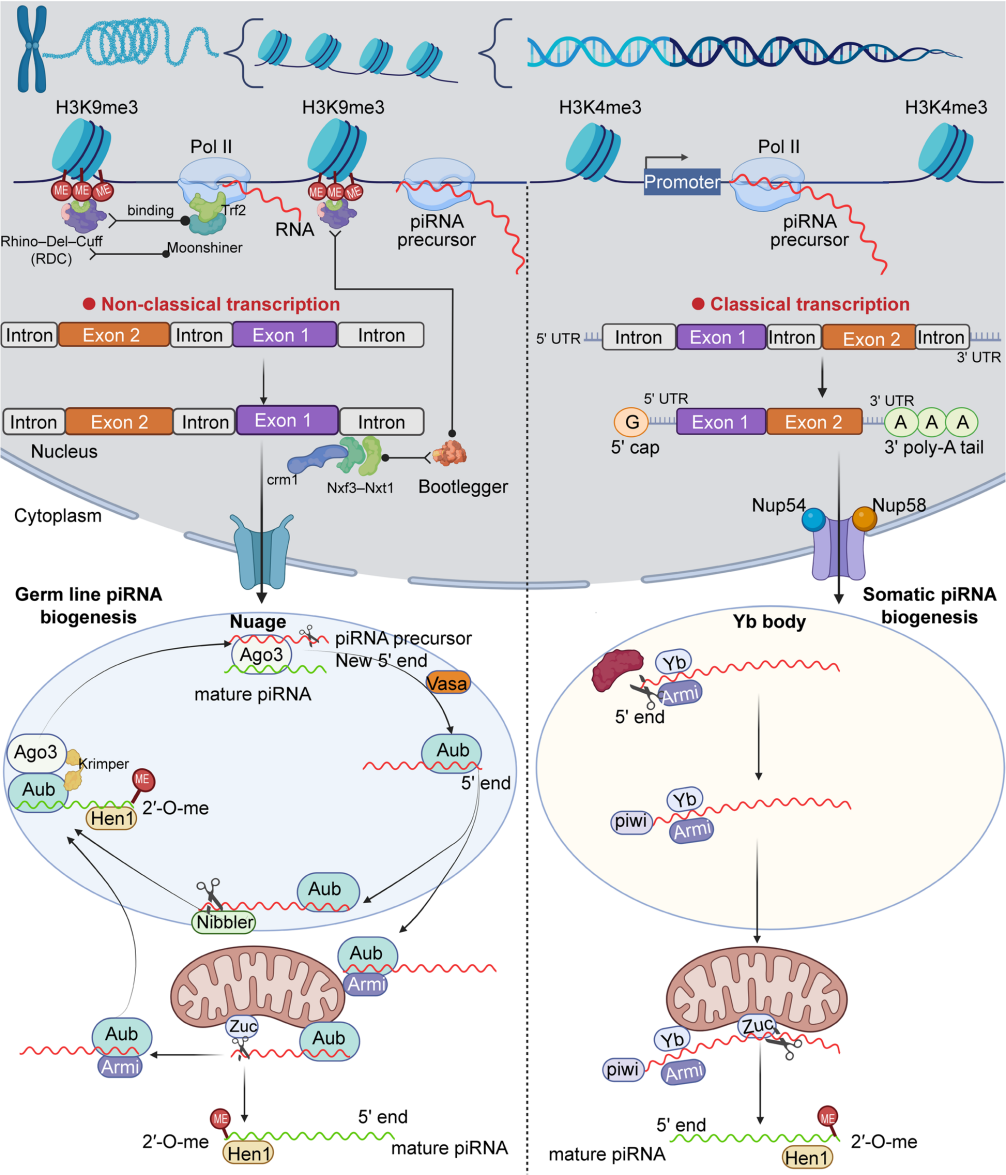
Left: piRNA cluster formation and germ line piRNA biogenesis. Rhino forms a terpolymer complex with Deadlock and Cutoff called RDC, and recognizes H3K9me3 on the piRNA cluster. First, RDC recruits Moonshiner and Trf2 to initiate transcription, and upon completion of transcription, piRNA cluster precursor is formed. Then RDC complex achieves the binding of NXF3-NXT1 complex by recruiting adapter protein Bootlegger (boot). Finally, Nxf3 binds Crm1 to mediate the transfer of piRNA precursor transcription out of the nucleus and localization to Nuage in the cytoplasm. In nuage, the 3 ‘end of the loaded piRNA precursor transcribed on Aub is trimmed by Nibbler, and then the end is subjected to 2’ -O-methylation by the methyltransferase Hen1 to become mature piRNAs. Finally, Krimper realized the transition of mature piRNA. In the mitochondria, Armi transfers the piRNA precursor transcript to the mitochondrial outer membrane, where it is cleaved by Zuc to produce the first piRNA. And then it is transported back to nuage. The rest of the piRNA precursor transcripts are repeatedly bound by PIWI and cleaved by Zuc, generating a string of PIWI-loaded piRNAs that are methylated by Hen1. Right: piRNA cluster formation and ovarian somatic cells piRNA biogenesis. The single-stranded piRNA cluster in ovarian cells contains a promoter itself, whose transcription initiation depends on transcription factor Pol II. The piRNA precursor transcripts form standard mRNA structures in the nucleus by standard splicing, 5 ‘-end closure, and 3’ -end polyadenylation. The Nxf1-Nxt1 complex and nuclear porins Nup54 and Nup58 then mediate the export of these transcripts to the Yb body. In Yb bodies, PIWI binds to the 5 ‘end of the flam precursor transcript and connects to Armi, then translocate to the mitochondrial outer membrane, where zuc cleavage produces a new 3’ end, which is finally subjected by Hen-1 to produce mature piRNA

Homeostasis of an organism primarily depends on homeostasis and regulation of the genome, critically influencing development and balance [28, 29]. The maintenance of this homeostasis is mainly achieved by diverse molecular events, in which RNA silencing plays a significant role in eukaryotes [30, 31]. Argonaute proteins are composed of the AGO and the PIWI family. Among them, PIWI proteins are mainly expressed in germ cells and form specific RNA-induced silencing complex (RISC) with piRNAs, collectively referred to as piRISC [10, 32, 33], which primarily regulate the expression and transposition of transposons in the genome and affect the activities. Here, we mainly review the biogenesis and functional mechanisms of piRNAs (Fig. 2).

### piRNA cluster: the origin of piRNAs

Large amounts of piRNA, known as “piRNA clusters”, are generated from specific genomic loci, which are identified as genomic regions ranging from few hundred to several thousand kilobases [34, 35]. piRNA clusters are first transcribed by RNA polymerase II (Pol II) into long noncoding transcripts and piRNA precursors in the nucleus and then enter the cytoplasm for further processing and modification [36]. The *Drosophila melanogaster* model is the earliest and most mature model for piRNA cluster research [37]. Therefore, in this section, we will use the *Drosophila* model as a template to introduce the piRNA cluster generation in detail from the main production sites, the key enzymes required, and the main processes.

In germ cells, the piRNA cluster is bidirectional, lacking a well-defined promoter region. Hence transcription regulation mostly depends on the histone H3 Lys9 (H3K9) trimethylation mark present in *D. Melanogaster* female germ cells [38, 39]. Rhino, a heterochromatin protein 1 (HP1) family protein, first binds to this mark and simultaneously recruits Deadlock and Cutoff proteins to form a Rhino-Deadlock -Cutoff (RDC) complex [40, 41]. RDC activates the transcription of piRNA clusters through irregular mechanisms. Specifically, Deadlock recruits the transcription initiation factor Moonshiner, which induces Pol II-mediated transcription by binding to TATA box-binding protein-associated Factor 2 (Trf2) [42, 43]. Cutoff binds the new piRNA precursors to protect them from degradation by blocking splicing and polyadenylation [44, 45]. Thus, the absence of splicing or polyadenylation is one of the most striking features of piRNA precursor transcripts [42, 46]. Finally, the piRNA prerequisite transcript passes through the nuclear pore through the nuclear output adapter complex Nxf3-Nxt1 [47]. The resulting piRNA precursors are then transported to the “Nuage” region in the cytoplasm and further processed into mature piRNAs [48, 49].

Another classic type comprises the unidirectional piRNA cluster with promoters in *Drosophila melanogaster* ovary cells [50]. The Flamenco (flam) locus is the most representative one, with no H3K9 trimethylation mark but has a specific independent well-defined promoter, also transcribed by Pol II [51–53]. In this case, the resulting transcripts are spliced and processed in the nucleus to form characteristic RNA precursors (5’ caps and 3’ poly(A) tails) [54]. The piRNA precursors are then transported to the perinuclear RNA particle “Yb body” for further processing [55]. The classical output complex NXF1-NXT1 is instrumental in the intracellular transport of piRNA precursors from the nucleus to the cytoplasm, necessary for the subsequent localization to the Yb body [56]. The nucleoporins Nup54 and Nup58 specifically transfer flam precursor transcripts from the nucleus and link to the Yb body to participate in the precise localization of piRNA precursor [54].

### Biosynthesis of piRNA: two classical pathways

Upon export to the cytoplasm, the piRNA precursor transcripts are localized to ribonucleoprotein particles near the nucleus, such as Yb body and Nuage, and then processed into piRNA with mature functions [57, 58].

In *D. melanogaster*, the biogenesis of piRNA in germ cells is processed through the ping-pong cycle, mainly in a specific region in the cytoplasm called Nuage and partly in the outer membrane of mitochondria [59]. The transport of piRNA precursor transcripts in this process primarily depends on the exchange of two cytoplasmic PIWI proteins: Aubergine (Aub) and Argonaute 3 (Ago3) [60–62].

The ping-pong cycle is initiated by the Aub, recognizing complementary piRNA precursor transcripts and slices from the 5’ end between nucleotides 10 and 11 to generate a new 5’ end [63]. Ago3 then interacts with a 5 ‘-monophosphorylated fragment, cleaved in Nuage by an endonuclease of Aub or Ago3 or by Zucchini (zuc) on the mitochondrial outer membrane to produce a 3’ end [64]. Zuc-dependent cleavage produces piRNAs of the final size, and the 3 ‘to 5’ exonuclease Nibbler, which forms the 3 ‘end of piRNAs by trimming the 3’ end to its final length, is also involved in AGO3-mediated piRNA generation [65, 66]. Methyltransferase Hen1 then methylates the 3’ end with 2’-O- methylation to produce the mature piRNA, which is protected from degradation [67, 68]. In addition, the Aub of the loaded dimethylated piRNA and the Ago3 in the unloaded state are specifically identified and combined by Krimper to ensure a sustainable ping-pong cycle [64]. The following process is mediated by the RNA helicase Armitage (Armi) and is initiated in the mitochondria. First, Aub bound to the 5 ‘-monophosphorylated piRNA precursor mutually and linked to Armi, which transfers the complex to the outer membrane of the mitochondria [69]. Here, it is cleaved by Zuc to produce a new 3’ end. The remaining piRNA precursor transcripts are bound by Piwi at the 5’ end and then continue to be cleaved by Zuc to produce a series of piRNAs ranging in length from 24 to 32 nucleotides that are eventually transferred to the nucleus [70].

The lack of expression of Aub and Ago3 in the somatic cells of Drosophila ovaries causes the piRNA precursor transcripts to be only dependent on the cleavage by Zuc [71]. Flam-piRNA precursor transcripts are first accumulated with protein Yb, then transferred by Armi to the mitochondrial outer membrane, where they are cleaved by Zuc to produce a new 3’ end [72]. Eventually, they are treated by 2’ -O - methylation of Hen1 to produce the mature piRNA.

### Cellular functions of PIWI–piRNA complexes

The interaction between piRNAs and PIWI proteins prevents transposable element (TE) activity and regulates the encoded mRNAs through different mechanisms [73]. This section primarily describes the piRNA-mediated transcriptional silencing of TE and the functional role of piRNA/PIWI protein complexes in regulating protein-coding genes.

### Silencing of transposable element

Transposon activity must be maintained through integration into germ cell DNA. In contrast, silencing of a transposon leads to inactivation of the protein encoded by the transposon as well as transposon death demise. The PIWI-piRNA complex was initially discovered to silence transposable elements (TEs) and maintain the stability and integrity of the germ cell genome [74, 75]. Different complexes formed by PIWI proteins and piRNAs silence TEs at the transcriptional and post-transcriptional levels, respectively [76]. Cytoplasmic PIWI proteins, such as Aub and Ago3 in Drosophila and MIWI and MILI in mice, primarily slice TE transcripts after transcription via piRNA targeting [77]. Nuclear PIWI proteins, including those in Drosophila PIWI proteins and mouse MIWI2, were incorporated into the nucleus after being loaded with phased piRNAs [78, 79]. Nuclear PIWI proteins bind to nascent RNAs through guiding piRNAs at the site of TE transcription and recruiting cofactors for transcriptional silencing [80].

### Regulation of protein-coding genes

In addition to TE silencing, piRNA regulates protein-coding genes during reproductive development; first discovered in Drosophila embryos, where TE-derived piRNA directed Aub to Nanos mRNA to subsequently mediated its deadenylation by recruiting deadenylate complexes [19, 26, 81]. Similarly, Aub loaded with piRNA can also directly degrade maternal mRNA in somatic cells in the early embryos of Drosophila [82]. Aub also interacts with poly(A) binding protein PABP and eIF3 subunits to activate the translation initiation of Nanos mRNA in embryonic reproductive cells, highlighting the crucial role of the evolutionary conservation of PIWI-piRNA complexes in translation activation [60]. These findings reflect the diversity of piRNA-regulated protein-coding genes, but the mechanisms behind these regulations remain to be fully understood.

### Roles of piRNA/PIWI complexes in regulating biological processes in human diseases

With increasing research on piRNA/piwi protein, dozens of abnormally expressed complexes are found to be related to the occurrence and development of diseases [15, 83, 84]. The mechanisms underlying their function include activating a pathway or target. These changes alter the initial homeostasis in cells and tissues to cause abnormal regulation of piRNA/PIWI proteins, which include sustained proliferation signaling, immune microenvironment remodeling, angiogenesis induction, apoptotic resistance, activation invasion and metastasis, and cell energy metabolism [85–89]. We classified the reported abnormalities according to their roles in disease and elaborated on their functions to elucidate the mechanisms fueling the disease-causing functions of the piRNA/PIWI proteins (Table 1).

**Table 1:** Roles of piRNAs/PIWI complex in biological processes in human diseases

Regulatory mechanisms	piRNA/PIWI	Expression	Diseases	Targets/signaling pathway	In vitro	In vivo	Ref.
Abnormal cell cycle and Resistance to apoptosis	piR-823	up	Colorectal cancer	HSF1	Promote cell cycle and inhibit cell apoptosis	NA	[90]
	piR-017061	down	Pancreatic cancer	ERK signaling pathway	Inhibit cell growth and induce apoptosis	Attenuate the volumes and weights of the xenografts in vivo	[91]
	piR-36,712	down	Breast cancer	P53 signaling pathway	Suppress cell proliferation, migration and invasion	Lower tumor growth rate	[92]
	piR-004800	up	Multiple Myeloma	PI3K/Akt/mTOR Pathway	Promote proliferation and inhibit apoptosis and autophagy	Promote MM proliferation	[93]
	piR-8041	down	Glioblastoma	NA	Induce cell cycle arrest and apoptosis	Restrict tumor growth temporarily	[94]
	PIWIL1	up	Glioblastoma	CDKN1B and CCND2	PIWIL1 knockdown causes cell cycle arrest, and induces apoptosis and promotes senescence.	PIWIL1 Knockdown reduces Tumor Growth and improves survival	[95]
	piR-30,188/PIWIL3	down	Glioblastoma	OIP5-AS1/miR-367/CEBPA/TRAF4 pathway	Suppress tumor cell proliferation, invasion, and migration, and promotes apoptosis	Restrain tumor growth and prolong nude mice survival	[96]
	piR-651	up	Non-small cell lung carcinoma	Cyclin D1 and CDK4	Promote cell cycle progression in G2/M phase	Promote tumor growth	[97]
	piR-651	up	Non-small cell lung carcinoma	Mitochondria-dependent pathway	Promote cell proliferation in a time-dependent manner and inhibits apoptosis, Promote NSCLC cell migration and invasion	NA	[98]
	PIWIL2	up	Esophageal squamous cell carcinoma	NF-κB signaling pathway	Promote cell proliferation, inhibit apoptosis, and activate autophagy	Promote tumor growth	[99]
Aberrant proliferative signaling pathways	piR-31,115	up	Clear cell renal cell carcinoma	PI3K/AKT Pathway	Promote cell proliferation, invasion and metastasis	NA	[100]
	piR-001773 and piR-017184	up	Prostate cancer	PI3K/AKT Pathway	Promote cell growth, invasion, and migration	Promote PCa cell growth, invasion, and migration	[101]
	piRNA-823	up	Luminal breast cancer	Wnt/β-catenin signaling pathway	Acquire stem cell-like properties, promote cell proliferation	Promote tumor growth	[102]
	piR-55,490	down	Lung cancer	Akt/mTOR pathway	Reduce the proliferation	Suppress the growth of xenografts	[103]
Activation of invasion and metastasis	piR-57,125	down	Clear cell renal cell carcinoma	AKT/ERK signaling pathway	Restrain cell metastasis	Knockdown of piR-57,125 promotes ccRCC cells metastasis	[104]
	piR-19,166	down	Prostate carcinoma	CTTN/MT1-MMP/MMP2/MMP9 signaling pathway	Hinder cell migration	Reduce the number and size of metastatic nodules	[105]
	piR-39,980	up	Osteosarcoma	MMP2	Induce the proliferation and enhance the invasion and metastasis	NA	[106]
	piR-54,265	up	Colorectal cancer	MMP2 and MMP9	Promote proliferation, migration and invasion	Promote tumor growth, spread and metastasis.	[107]
	PIWIL2	up	Prostate cancer	MMP9	PIWIL2 knockdown weakened tumor cell invasion and migration	NA	[108]
	PIWIL1	up	Pancreatic cancer	Pinin	Increase the proliferation, invasion and metastasis	Promote growth and metastasis of xenografts	[109]
	piRNA-823	up	Multiple myeloma	VEGF and IL-6	Promote angiogenesis and invasion	Promote the growth of xenograft tumors	[110]
	piRNA-823	up	Multiple myeloma	VEGF	Promote proangiogenic activity	Promote the growth of xenograft tumors	[111]
EMT	piR-17,560	up	Breast cancer	FTO/ZEB1 signaling pathway	Promote the proliferation, EMT and chemoresistance	Increase tumor volume under chemotherapy drug treatment	[112]
	piR-31,115	up	Clear cell renal cell carcinoma	NA	Promote migration and invasion	NA	[100]
	PIWIL1	up	Endometrial cancer	NA	Endow endometrial cancer [EC] cells with stem-like properties, such as tumor cell viability, migration, invasion, and sphere-forming activity.	Increase tumor growth	[113]
	piR-1037	up	Oral squamous cell carcinoma	NA	Enhance the motility	Increase tumor growth	[114]
Energy metabolism	piR-823	up	Colorectal cancer	PINK1-Parkin mediated mitophagy	Maintain mitochondrial function, dynamics, and quantity	Block mitophagy, increases tumor volume and tumor weight	[115]
	piR-hsa-211,106	down	Lung Adenocarcinoma	Pyruvate carboxylase	Attenuate cell proliferation and colony formation	Suppress tumor xenograft growth	[116]
	PIWIL2	up	Radiation-induced pulmonary fibrosis	Purine metabolism	Activate purine metabolism and promote the attenuation of RILF	Alleviate pulmonary fibrosis	[117]
	PIWIL1	up	Hepatocellular carcinoma	MLYCD	Induce fatty acid metabolism	Promote the tumor growth	[118]
DNA damage	piR-39,980	down	Fibrosarcoma	RRM2	Enhance apoptosis of fibrosarcoma cells induced by DOX treatment	NA	[119]
	PIWIL4	down	Fibrosarcoma	ROS accumulation	Reduce cell growth, migration and invasion	NA	[120]
	piR-31,470	up	Prostate cancer	GSTP1	Promote call proliferation	NA	[121]
Immune microenvironment	PIWIL4	up	HIV-1 Latency	SETDB1、HP1α/β/γ	Maintains HIV-1 latency in infected CD4 + T lymphocytes and inhibits viral replication	NA	[122]
	PIWIL1	up	Hepatocellular carcinoma	IL10	Inhibition of immune response in the tumor microenvironment	promotes the tumor growth	[118]
	piR-DQ551351	down	Prostate cancer	M2 macrophages polarization	NA	inhibit tumor growth	[123]
	piR-DQ551309	up	Prostate cancer	M2 macrophages polarization	NA	inhibit tumor growth	[123]

### Epigenetic modifications mediated by piRNA/PIWI complex

**Fig. 3:**
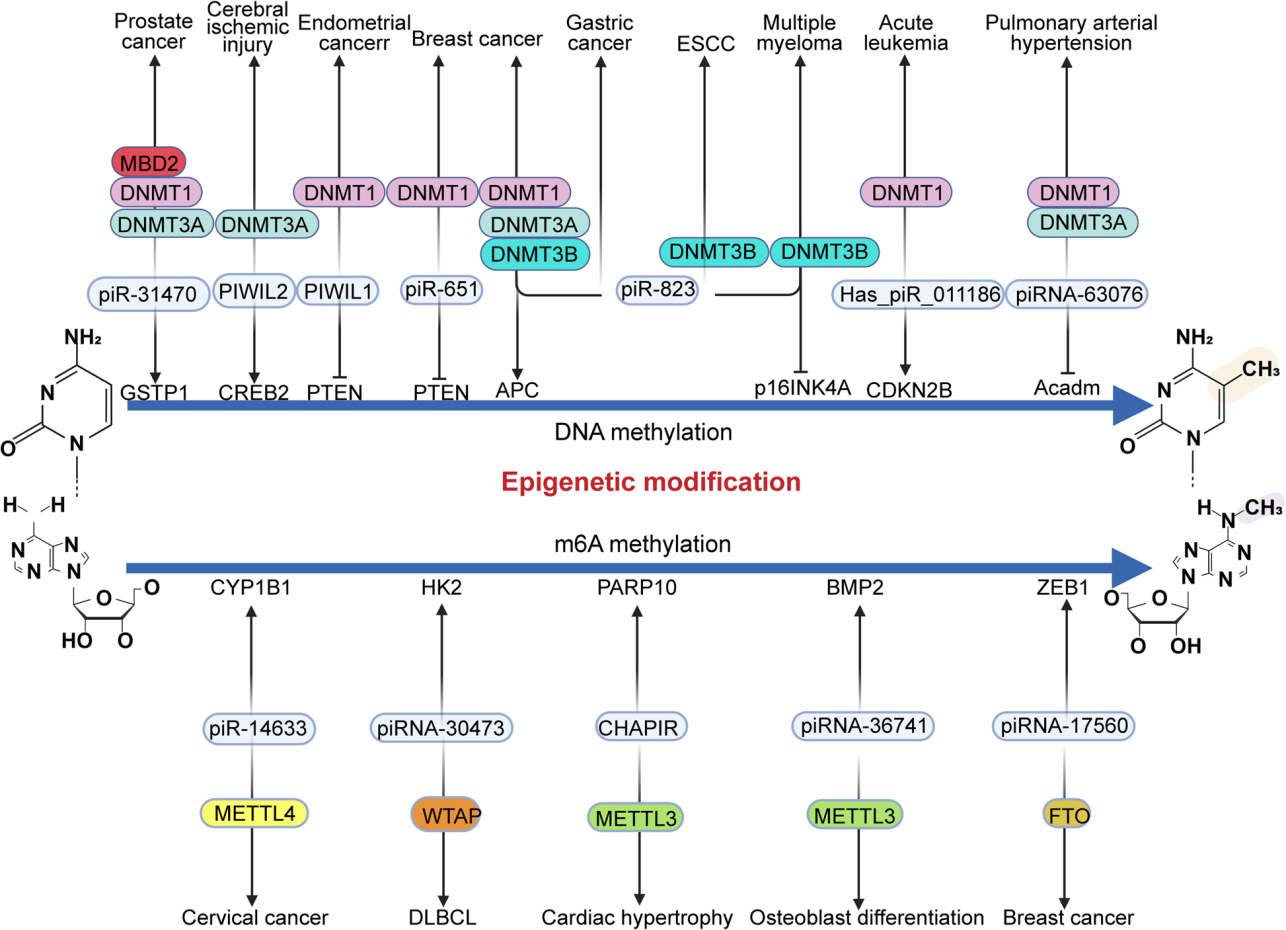
Differential methylation modification in different diseases. The top half is the DNA methylation mediated by piRNA and PIWI proteins in different diseases. In this section, the top part are the corresponding disease types, the middle part are the enzymes mediating the methylation modification, and the bottom part are the targets of the ultimate effect. The bottom half is the m6A methylation modification carried out by piRNA/PIWI protein in the diseases. In this section, the bottom part are the corresponding disease types, the middle color blocks are the enzymes whose methylation modification is performed, and the top part are the targets of the final effect.

Existing research suggests that piRNAs primarily mediate epigenetic changes through abnormal methylation and histone modification in somatic cells, eventually causing the diseases [124, 125] (Fig. 3).

DNA methylation is critical to epigenetic modification, primarily by silencing transposons and other repeating elements [126, 127]. Four major types of DNA methyltransferases (DNMTS) regulate DNA methylation and its patterns [128]. In this section, we mainly summarize the biological roles of epigenetic modifications mediated by piRNA/PIWI proteins in normal functions and human diseases (Table 2).

**Table 2:** Roles of piRNA/PIWI proteins in epigenetic modifications

Type of methylation	piRNA/piwi	Expression	Diseases	Mechanism	In vitro	In vivo	Ref.
DNA methylation	piRNA-823	up	Multiple Myeloma	Upregulate the expression of DNMT3B, promote global DNA methylation and decrease tumor suppressor p16INK4A	Promote the cell stemness of MMSCs and lead to abnormal proliferation, anti-apoptosis and tumorigenesis.	Enhance MM growth, tumor burden and angiogenesis	[129] [111]
DNA methylation	piRNA-823	up	ESCC	NA	NA	NA	[130]
DNA methylation	piRNA-823	up	Breast Cancer	Upregulate the expression of DNMT1, DNMT3A, and DNMT3B, then promote methylation of the APC promoter, and mediates Wnt pathway activation	Promoted cell proliferation and cell Stemness	Promote tumor growth	[131]
DNA methylation	piRNA-823	down	Gastric Cancer	Reduce tumor suppressor genes methylation, and then causes cells aberrant “stem-like” state	Suppress tumor cell growth	Reduce tumor weight	[132]
DNA methylation	piR_011186	up	Acute Leukemia	Promote DNA methylation of CDKN2B promoter	Promote cell cycle progression, and reduce apoptosis	NA	[133]
DNA methylation	piR-31,470	up	Prostate Cancer	Recruits DNMT1, DNMT3α and MBD2 to induce hypermethylation of GSTP1	Promote cell proliferation	NA	[134]
DNA methylation	piR-651	up	Breast Cancer	Increases PTEN promoter region methylation and restrain its expression	Promote cell proliferation and invasion, and inhibit apoptosis	NA	[135]
DNA methylation	piRNA-63,076	up	Pulmonary arterial hypertension	Upregulate levels of DNMT1 and DNMT3A, increase the methylation of Acadm promoter and result in the inhibition	Increase the proliferation of pulmonary arterial smooth muscle cells	NA	[136]
DNA hypomethylation	PIWIL1	up	Endometrial Cancer	PIWIL1 promoter hypomethylation	Promote cell growth	NA	[137]
DNA hypermethylation	PIWIL1	up	Endometrial Cancer	Induce PTEN hypermethylation and inhibit the expression	NA	NA	[138]
DNA methylation	PIWIL2	down	Cerebral ischemic injury	PIWIL2 knockdown leads to a significant reduction in DNMT3A, which in turn will eliminate CREB2 DNA methylation and increase CREB2 expression	Prevent dentate spine loss	NA	[139]
m6A methylation	piRNA-30,473	up	DLBCL	WTAP promotes the stable expression of its downstream target HK2 by increasing its m6A level	Promote cell proliferation	Promote tumor growth	[140]
m6A methylation	piRNA-14,633	up	Cervical Cancer	Highly expressed METTL14 promotes m6A methylation of CYP1B1	Promote cell proliferation, migration and invasion	Promote cancer growth	[141]
m6A methylation	piRNA-17,560	up	Breast Cancer	Strengthen ZEB1 transcripts stability and expression by decreasing m6A RNA methylation	Promote the proliferation, EMT and chemoresistance	Increase drug resistance and tumor volume after chemotherapy	[112]
m6A methylation	piRNA-36,741	up	Osteoblast differentiation	piR-36,741 trapped METTL3 to prevent it from binding to BMP2 mRNA, thereby reducing m6A methylation of BMP2	Promote osteogenic differentiation	Alleviate ovariectomy-induced osteoporosis in mice	[121]
m6A methylation	piR-141,981	up	Cardiac hypertrophy	CHAPIR captures METTL3 to prevent its binding to PARP10 mRNA and reduces the m6A methylation of PARP10	Promote cardiomyocyte hypertrophy	Promote cardiomyocyte hypertrophy	[142]

piR-823 regulates the methylation of cancer stem cells by regulating DNMT1, DNMT3A, and DNMT3B. Ai et al. demonstrated that silencing of piRNA-823 reduced global DNA methylation induced by myeloid-derived suppressor cells (MDSCs) and reduced the stemness of multiple myeloma stem cells (MMSCs) maintained by MDSC, reducing tumor burden and angiogenesis in vivo [111, 129]. In addition, Yan et al. further explored the specific mechanism of piR-823 in MM, where the expression of piR-823 positively correlated with DNMT3A and DNMT3B. The high expression of DNMTs induces p16INK4A hypermethylation, leading to the loss of many protein-encoding tumor-suppressor genes (TSGs) [111]. piR-823 also induces aberrant DNA methylation through DNMT3B and has a tumorigenic role in ESCC [130]. Ping et al. reported piR-823 to be highly expressed in breast cancer cells and positively correlated with the expression of DNMT1, DNMT3A, and DNMT3B. Further research showed that the upregulation of piR-823 was positively correlated with m5C, the methylation target in the adenomatous polyposis coli (APC) promoter region. Meanwhile, aberrant APC promoter methylation also mediates Wnt signaling pathway activation [131]. Interestingly, piR-823 confer a tumor suppressor role in gastric cancer, these aberrant expression of piRNAs may directly or indirectly suppress tumor suppressor genes, resulting in an abnormal “stem-like” cellular state, inducing tumorigenesis [132].

In leukemia cells, upregulated hsa_piR_011186 binds to DNMT1 and inhibits CDKN2B gene expression by increasing DNA methylation at the CDKN2 promoter, inducing cell cycle progression and reducing cell apoptosis [133]. piR-651 is highly expressed in breast cancer tissues and promotes the expression of oncogenes such as MDM2, CKD4, and CyclinD1. Upregulated piR-651 leads to an increase in DNMT1, which binds to the promoter of Phosphatase and tensin homolog (PTEN), increases its methylation and inhibits its expression [135]. Zhang et al. reported that piR-31,470/PIWIL4 complex induces hypermethylation of glutathione S-transferase pi 1 (GSTP1) by recruiting DNMT1, DNMT3A, and MBD2, resulting in lower expression of GSTP1, which renders the cells more susceptible to oxidative stress, making them more easily acquire the tumorigenic phenotype [134]. piR-63,076 is highly expressed in hypoxic pulmonary arteries, upregulating DNMT1 and DNMT3a, which subsequently increases the methylation of the Acadm promoter and leads to the inhibition of its expression under hypoxia [136].

PIWIL1 is silenced in normal endometrium and is reactivated and carcinogenic in endometrial cancer. In endometrial cancer cells, estrogen E2 induces ERα to bind to the PIWIL1 promoter, leading to hypomethylation of the promoter, which then upregulates PIWIL1 transcription [137]. Studies have also shown that PIWIL1 induces hypermethylation of PTEN by increasing the expression of DNMT1 and inhibiting the tumor suppressor PTEN to promote cancer progression [138]. In addition, silencing PIWIL2 plays a protective role in transient global cerebral ischemia (tGCI) tolerance induced by hypoxic postconditioning (HPC). Zhan et al. found that HPC downregulated PIWIL2 expression in CA1. Low expression of PIWIL2 decreased the expression of apoptosis-related proteins and exerted a neuroprotective effect. CREB2 is a crucial regulator of neuronal plasticity and memory [143, 144]. PIWIL2 damages neurons after cerebral ischemia by regulating DNA methylation in the CpG island of the CREB2 promoter. Moreover, HPC-induced PIWIL2 downregulation following tGCI leads to a significant reduction in DNMT3A level, eliminating the TGCI-induced increase in DNA methylation of CREB2 [139].

Recent studies have also shown that piRNAs contribute to cancer development and progression by regulating abnormal m6A methylation modification [145–147]. m6A methylation is the most common and abundant internal post-transcriptional modification [148, 149]. Evidence shows that m6A methylation significantly impacts RNA biosynthesis/catabolism and regulates the pathogenesis of various cancers [150, 151].

Previous studies demonstrated that Wilms’ tumor 1-associating protein (WTAP) (an m6A methyltransferase) is required for m6A methyltransferase activity in vivo [152]. In diffuse large B-cell lymphoma (DLBCL), Han et al. showed that piRNA-30,473 increases WTAP mRNA expression by binding to its 3’UTR, reducing WTAP mRNA degradation and enhancing its stability. The high expression of WTAP further promotes the stable expression of its downstream target HK2. Then, IGF2BP2 binds to the 5 ‘UTR region of HK2 to stabilize its methylated transcripts [140]. piRNA-14633 is highly expressed in cervical cancer, and the total abundance of m6A increases with its overexpression. Further studies showed that piRNA-14633 specifically binds METTL14 in cervical cancer cells and enhances its expression by improving mRNA stability. The high expression of METTL14 promotes cancer progression by promoting m6A methylation of its downstream target, CYP1B1 [141]. Senescent neutrophil-derived exosome piRNA-17560 increases binds to fat mass and obesity-associated protein (FTO), reduces its mRNA decay and enhances its stability in breast cancer. FTO is one of the vital m6A mRNA demethylation enzymes, the upregulation of which further enhances the strength and expression of ZEB1 transcripts by reducing the m6A methylation of mRNA [153, 154]. In addition, m6A reader protein YTHDF2 binds to ZEB1 transcripts to induce post-transcriptional regulation [155, 156]. In conclusion, YTHDF2 and FTO increase the stability and expression of ZEB1 mRNA, leading to drug resistance and high aggressiveness of breast cancer cells [112].

piRNA-mediated methylation also plays a role in normal physiological functions. In osteoblast differentiation, piRNA-36,741 and METTL3 are highly expressed, and their silencing inhibits the normal differentiation of osteoblasts. Mechanistically, piR-36,741 sequesters METTL3 to prevent its binding to BMP2 mRNA. The m6A modification level of BMP2 is then significantly increased, allowing YTHDF2 (m6A reader) to recognize and degrade the m6A methylated BMP2 transcript, resulting in decreased expression and reduction in osteogenic phenotype and matrix mineralization [121]. In addition, Gao initially found that cardiac-hypertrophy-associated piRNA (CHAPIR) deficiency ameliorated cardiac hypertrophy and remodeling. Mechanistically, the CHAPIR-PIWIL4 complex binds to METTL3, reduces its activity to prevent the binding to PARP10 mRNA, and reduces its m6A methylation, thereby avoiding recognition and degradation by YTHDF2. PARP10 then accelerates cardiac hypertrophy by blocking GSK3β activity and weakening NFATC4 phosphorylation [142].

### Abnormal cell cycle and resistance to apoptosis

Apoptosis is representative of programmed cell death, the inhibition of which leads to uncontrolled cell division [157, 158]. An abnormal cell cycle further leads to uncontrolled malignant proliferation, causing cancer [159, 160].

Cyclin D1 and CDK4 are essential regulators of the G1 phase of the cell cycle [161, 162]. Silencing piR-823 via an anti-sense sequence inhibits Cyclin D1 and CDK4 expression, blocking the cell cycle by inducing G1 phase arrest and inhibiting G1/S transition [90]. Moreover, piR-017061 upregulation induces apoptosis of pancreatic cells and significantly reduces the tumor volume and weight in vivo. Notably, overexpression of piR-017061 significantly reduced the expression of Bcl-2, simultaneously increasing the cleaved Caspase3 and decreasing p-ERK levels, suggesting the critical role of ERK signaling in apoptosis regulation [91]. As a tumor suppressor, piR-36,712 suppresses the malignant phenotype. Mechanistically, the interaction of piR-36,712 with SEPW1P reduces SEPW1 expression and function, with a subsequent increase in p53 levels and resulting in G1 cell cycle arrest. These results suggest that piR-36,712 increases the expression of p53 to exert its tumor-suppressive effects [92]. Overexpression of piR-004800 promotes proliferation and inhibits apoptosis in multiple myeloma (MM) cells. Mechanistically, the piR-004800 downregulation decreases the expression of anti-apoptotic genes, such as Bcl2 and caspase-3, and increases the expression of related pro-apoptotic proteins BAD, cleaved PARP, and cleaved caspase-3 [93]. After piR-8041 overexpression, glioma cells were arrested at the G0/G1 checkpoint, with decreased entry to the S-phase. However, the specific signaling pathways or proteins regulating the cell cycle have not been discovered and confirmed [94]. PIWIL1 knockdown induces the expression of CDKN1B and CCND2, resulting in upregulation of the cyclin-dependent kinase inhibitor p27, which regulates G1 progression by inhibiting cyclin activity. In addition, PIWIL1 knockdown inhibits MCL1 expression, promoting apoptosis and senescence [95]. PIWIL3 and piR-30,188 complex bind to carcinoma-related OIP5-AS1 to reduce its expression, reversing its inhibitory effect on its downstream tumor suppressor target miR-367-3p. The highly expressed miR-367-3p, in turn, binds and reduces CEBPA expression, inhibiting its binding to the downstream TRAF4 promoter and weakening TRAK4 activation. Finally, it inhibits the malignant growth of glioma and promotes apoptosis [96].

Overexpression of piR-651 promoted G cell cycle progression by inducing cyclin D1 and CDK4, and cells overexpressing piR-651 had a greater ability to form colonies. In vivo experiments showed that overexpression of piR-651 resulted in greater tumor volume and weight [97]. In addition, piR-651 also exerted its anti-apoptotic effect by affecting mitochondria-related proteins, such as increasing caspase-3-p17, Bax, and cleaved-PARP-1 and decreasing Bcl-2 [98]. PIWIL2 promotes cancer progression by inhibiting apoptosis primarily through IκB kinase (IKK)/IκB/NF-κB pathway in esophageal cell squamous carcinoma (ESCC). PIWIL2 binds to IKKα and IKKβ in the cytoplasm, significantly upregulating the total protein and phosphorylation levels of IKKα/β. Phosphorylated IKK induces IκB phosphorylation and NF-κB release for nuclear translocation and apoptosis inhibition. In addition, PIWIL2 competitively inhibits IKKβ binding to TSC1, inactivating mTORC1 and promoting ULK1 phosphorylation and initiating autophagy [99].

### Aberrant proliferative signaling pathways

Uncontrolled malignant cell proliferation is one of the most prominent features of cancer, causing tumor growth [163, 164]. Mechanistically, intracellular and extracellular signaling molecules regulate the orderly proliferation and apoptosis of cells to regulate regular operations [165–168].

Oncogenic piR-31,115 promotes the unrestricted proliferation of ccRCC cells. In contrast, silencing piR-31,115 inhibits cell proliferation and significantly reduces AKT phosphorylation and PI3K expression, potentially promoting proliferation by activating EMT and PI3K/AKT signaling pathways [100]. piR-001773/piR-017184 and PCDH9 mRNA co-exist in the PIWIL4 complex and decrease its expression. In PCa cells, PCDH9 acts as a competitive substrate for PI3K binding and hinders the catalytic PIP3 synthesis reaction. Thus, PCDH9 silencing leads to increased AKT phosphorylation and activated AKT contributes to increased cell proliferation and inhibition of apoptosis [101]. Overexpression of piR-823 activates the Wnt/β-catenin pathway, leading to the malignant proliferation of breast cancer cells [131]. piR-55,490 binds to the 3’UTR of mTOR mRNA and degrades it, preventing the activation of the AKT/mTOR signaling pathway [103].

### Invasion and metastasis activation

Tumor cell malignant behaviors, invasion, and metastasis are closely related to the poor prognosis of patients, causing the death of patients with different types of cancer [169–171]. Most tumors miss the best treatment opportunities due to early metastasis, and patients with the initially diagnosed metastatic disease have the worst 5-year survival rate compared with individuals with localized disease [172–174].

Ding et al. showed that piR-57,125 upregulation specifically inhibits chemokine (C-C motif) ligand 3 (CCL3) expression. Mechanistically, piR-57,125 suppresses invasion and metastasis by inhibiting CCL3 and its downstream AKT/ERK signaling pathway activation [104]. piR-19,166 overexpression significantly hinders the migration of PCa cells, while it’s silencing significantly promotes their migration. Mechanistically, piR-19,166 inhibited the direct downstream target CTTN expression and the matrix metalloproteinases (MMP) signaling pathway, and then reducing the invasion and metastasis of PCa cells [105]. Overexpression of MMPs plays an important role in tumor invasion and metastasis, and MMP-2 has been characterized as the most potential therapy target [175, 176]. The highly expressed piR-39,980 directly reduces the expression of the tumor suppressor gene SERPINB1, relieving its inhibitory effect on the downstream target MMP-2. High MMP-2 levels significantly induce osteosarcoma cell proliferation, invasion, and metastasis [106]. piR-54,265 binds to PIWIL2 and promotes the formation of PIWIL2/STAT3/p-SRC complex. Upregulated piR-54,265 and PIWIL2 increase the expression of downstream targets MMP2 and MMP9 by promoting the activation of the STAT3 signaling pathway, enhancing the invasion and metastasis ability of CRC cells [107]. Knockdown of PIWIL2 reduces the expression of MM9 in prostate cancer cells, thereby reducing invasion and migration ability [108]. The high expression of PIWIL1 significantly enhances the invasion and metastasis of pancreatic ductal adenocarcinoma (PDAC) cells in vitro and in vivo. PIWIL1 activated the APC/ C-Ub pathway, and further drives the proteolysis of adhesion protein Pinin, enhancing PDAC metastasis [109].

The imbalance between hypoxia-induced overexpression of pro-angiogenic factor (VEGF) and anti-angiogenic factors leads to angiogenesis in the tumor microenvironment, further aggravating the proliferation and spread of cancer cells [177–180]. piR-823 overexpression in MM cells transmits signals to the tumor microenvironment (TME) via extracellular vesicles (EVs) to increase the expression levels of VEGF and IL-6 in endothelial cells, inducing angiogenesis and increasing tumor cells’ invasion and metastasis [110]. As a negative regulator of VEGF expression, overexpression of p16INK4A inhibits VEGF. Overexpressed piR-823 inhibits the expression of p16INK4A through methylation, thus promoting VEGF-mediated angiogenesis [111].

### Epithelial-mesenchymal transition (EMT)

Epithelial-mesenchymal transition (EMT) is the morphological transformation associated with the aggressiveness, metastasis, and drug resistance of malignant cells [181–184]. EMT is characterized by upregulated mesenchymal markers (N- cadherin and vimentin) and down-regulated epithelial markers (E‐ cadherin) [185, 186].

In breast cancer, senile neutrophils produce exosome piR-17,560 in a STAT3-dependent manner, which binds to FTO and increases ZEB1 expression by reducing m6A methylation, promoting chemotherapy resistance and EMT in BC cells [112]. piRNA-31,115 downregulation leads to the upregulation of E-cadherin, vimentin, decreased snail, and inhibition of cell migration in vitro. This indicated that the knockdown of piR-31,115 inhibits EMT and reduces cancer cell invasion [100].

EMT and the induction of stem-like properties of cancer cells are associated with solid tumors. PIWIL1 upregulates Snail mRNA expression and promotes EMT. Endometrial cancer cells with low PIWIL1 expression have upregulated E-cadherin, while Vimentin and N-cadherin were down-regulated. In addition, the expression of Vimentin, CD44, and ALDH1 was significantly reduced in the low-expression group, while that of E-cadherin was higher [113]. In OSCC cells, piR-1037 targeting significantly inhibited cell migration and invasion, with upregulated expression of E-cadherin and decreased levels of the N-cadherin, suggesting that piR-1037 knockdown could inhibit EMT [114].

### DNA damage and repair

DNA molecules are the fundamental genetic materials. The structural and functional integrity of DNA is crucial to support normal life activities and stable species traits [187, 188]. Endogenous or exogenous stress causes cells to produce various DNA damage, causing corresponding biological changes, such as carcinogenesis and abnormal cell apoptosis [189–192].

piR-39,980 causes apoptosis by increasing doxorubicin (DOX) accumulation. Mechanically, co-treatment of piR-39,980 mimics and DOX increased DNA fragmentation and γH2AX accumulation, which were mainly dependent on the regulation of the downstream targets RRM2 and CYP1A2 by piR-39,980. RRM2 is a catalytic subunit of ribonucleotide reductase (RR), which regulates DNA repair [193–195]. CYP1A2 is a phase I/II metabolic enzyme whose upregulation regulates resistance mechanisms by inactivating DOX [196, 197]. piR-39,980 inhibits the expression of RRM2 and CYP1A2 to prevent the natural resistance of fibrosarcoma to DOX by inhibiting DNA repair [119]. PIWIL4 can increase intracellular reactive oxygen species (ROS) accumulation by inhibiting the expression of antioxidant enzymes from causing DNA damage. The loss of antioxidant enzymes leads to excessive ROS generation in mitochondria [198–200]. Upon reaching a specific threshold, the mitochondrial membrane channel opens to release a large amount of ROS into the cytoplasm, causing cell and DNA damage [201, 202]. Overexpression of PIWIL4 induced G2/M arrest and apoptosis in fibrosarcoma cells, whereas knockdown of PIWIL4 enhanced cell proliferation, metastasis, and invasion [120]. piR-31,470, highly expressed in prostate cancer, forms a complex with PIWIL4 and maintains the hypermethylation of GSTP1, inactivating it and reducing its expression. GSTP1, an antioxidant factor with protective and antitumor functions, plays an important regulatory role in anti-oxidative damage, regulating cellular oxidative stress and promoting cytotoxic metabolism [102, 203, 204]. Epigenetic silencing of GSTP1 leads to exposure to long-term oxidative damage and increased accumulation of potentially mutagenic DNA adducts, ultimately causing prostate cancer [121].

### Regulation of metabolism

Mitochondria fulfills cellular energy requirements through ATP synthesis using oxygen to metabolize carbohydrates and fatty acids, which are essential for long-term cell survival [205–208]. piR-823 could increase the expression of mitochondrial chaperone HSP60 and 70, which could maintain mitochondrial stability and inhibit mitochondrial autophagy, thereby upregulating the quantity to meet the transitional capacity requirements in tumor cells [209–211]. The PINK1-Parkin pathway is necessary for mitophagy, leading to apoptosis [212, 213]. PINK1 recruits the E3 ubiquitin ligase, Parkin, from the cytosol to the mitochondria, phosphorylates it at Ser65, and induces mitophagy through ubiquitination [214, 215]. piR-823 promotes proteasome-mediated degradation of PINK1, causing mitophagy to maintain the high-energy metabolic state of tumor cells [115]. Pyruvate carboxylase (PC) is essential for islet glutathione synthesis and promotes its antioxidant capacity [216]. In addition, PC mediates glucose metabolism to meet the increased energy demands to facilitate the rapid proliferation of cancer cells [217–219]. piR-hsa-211,106 inhibits PC mRNA from directly interacting with its protein, impairing its normal function. PC knockdown attenuates cell proliferation and colony formation in vitro and suppresses tumor xenograft growth in vivo. In addition, PC inhibition significantly consumes the glutathione (GSH) pool, disturbs GSH homeostasis and reduces the ability to survive oxidative stress [116]. Overexpression of PIWIL1 in hepatocellular carcinoma cells significantly increases the intracellular ATP content to meet the needs of rapid cell proliferation. However, surprisingly, PIWIL1 expression failed to increase HCC cells’ glucose uptake and significantly increased the mitochondrial ROS. HCC cells increased intracellular free fatty acid consumption, leading to PIWIL1-mediated promotion of fatty acid metabolism to speed up energy production to meet the needs of rapid tumor growth [118]. PIWIL2 performs its anti-fibrotic function by reprogramming purine metabolism. PIWIL2 overexpression increases the levels of serine, nucleoside intermediates (AICAR), and nucleoside monophosphates (IMP and GMP), suggesting the probable requirement of purine biosynthesis in PIWIL2-driven remission of RILF. TGF-β1 is a critical factor in the development of radiation-induced pulmonary fibrosis [220–222]. Upregulation of PIWIL2 alone inhibits the radiation-induced increase of TGF-β1 and achieves the effect of anti-pulmonary fibrosis. Mycophenolate mofetil (MMF) reverses the inhibitory effect of Nrf2 or PIWIL2 on radiation-induced TGF-β1 expression. These results collectively suggest that PIWIL2 exerts its therapeutic impact on RILF by activating the purine metabolism axis [117].

### Immune microenvironment remodeling

HIV-1, as an integrated provirus, is hidden in the cellular genome and evades immune surveillance [223, 224]. The 5’ end of HIV-1 long terminal repeat (LTR) is a strong promoter driving viral gene transcription [225, 226]. PIWIL4 inhibits HIV-1 5’LTR-driven transcription by recruiting SETDB1, HP1α/β/γ, and other repressors [227]. Conversely, the depletion of PIWIL4 reactivates the virus from latent CD4 + T lymphocytes and initiates replication. These results reveal an association between nuclear PIWIL4 and provide new insight into the mechanisms underlying the mandatory latency of HIV [122]. PIWIL1 overexpression accelerates energy production by promoting fatty acid metabolism to meet the needs of rapid tumor growth in HCC cells. This can be achieved by improving the fatty acid β-oxidation (FAO). In addition, PIWIL1-induced metabolic transformation to FAO creates an immunosuppressive microenvironment, promoting cancer progression. Inhibition of FAO suppresses MDSC and activates CD8 + toxic T cells, inhibiting tumor growth. Overexpression of PIWIL1 leads to increased C3 secretion, transmitting the immunosuppressive signals generated by PIWIL1 to its microenvironment. In addition, C3 secretion also activates IL-10 production, inhibiting T-cell immunity. In conclusion, PIWIL1 critically plays a dual role in driving metabolic reprogramming and immune escape in HCC [118]. Macrophages undergo polarization to M1 and M2 types under the stimulation of different stimulus factors, among which M2-polarized macrophages are pro-tumorigenic, known as tumor-associated macrophages (TAM) [228–230]. During M2 polarization, DQ551351 expression was downregulated, while DQ551308 expression was upregulated. Further research showed that overexpression of piR-DQ551351 and low expression of piR-DQ551308 could promote the mRNA expression of M1-macrophage marker TNF-α to promote the antitumor activity. The tumor volume in the overexpression group of piR-DQ551351 is significantly smaller than that in the control group [123]. The results of this study may provide new ideas for the M1 polarization of macrophages in the tumor immune microenvironment, thereby inhibiting the immune escape of M2-polarized macrophages.

### Association of piRNAs/PIWI proteins with clinical characterization in diverse diseases

The dysregulated piRNA/PIWI proteins play an important role in the occurrence and development of diverse diseases. A growing body of evidence has confirmed the high correlation between piRNA/PIWI proteins and the clinical features of tumors and other non-cancer diseases. Here, we summarize the clinical relevance of piRNA/PIWI proteins in various systemic cancers and non-cancer diseases (Table 3).

**Table 3:** Association of dysregulated piRNAs/PIWI proteins with clinical characterization in cancers and non-cancer diseases

Scope of disease	Diseases	piRNA/PIWI protein	Roles in diseases	Clinical features	Ref.
Digestive system cancers	Oral squamous cell carcinoma	PIWIL2	Carcinogen	lymph node metastasis, histological type, and clinical stage	[231] [232]
	Oral squamous cell carcinoma	piR-1037	Carcinogen	chemotherapy resistance	[114]
	Esophageal cancer	PIWIL2	Carcinogen	long-term survival rate, pathological T-stage	[99]
	Esophageal cancer	piR-823	Carcinogen	lymph node metastasis and tumor size	[130]
	Gastric Cancer	piR-823	Tumor suppressor	NA	[132]
	Gastric Cancer	piR-1245	Carcinogen	tumor size, TNM stage, survival time	[233]
	Gastric Cancer	PIWIL1	Carcinogen	poor differentiation, high TNM stage	[234]
	Colorectal cancer	piR-020619 and piR-020450	Carcinogen	tumor size, early-stage	[235]
	Colorectal cancer	piR-54,265	Carcinogen	Stage I, high-grade intraepithelial neoplasia and tumor recurrence, TNM stage, progression-free survival and overall survival	[107] [236]
	Colorectal cancer	piR-24,000	Carcinogen	tumor differentiation, distant metastasis, TNM Stage	[237]
	Colorectal cancer	piR-823	Carcinogen	poor differentiation, stage III and IV, poorly differentiated	[90] [115] [238]
	Colorectal cancer	PIWIL1	Carcinogen	NA	[239] [240]
	Intrahepatic cholangiocarcinoma	PIWIL4	Tumor suppressor	overall survival	[241]
	Cholangiocarcinoma and Gallbladder carcinoma	piR-10,506,469 and piR-20,548,188	Carcinogen	NA	[242]
	Pancreatic cancer	PIWIL1	Carcinogen	tumor metastasis	[109]
	Pancreatic cancer	PIWIL2	Tumor suppressor	overall survival, T-stage and tumor aggressiveness	[243]
	Pancreatic cancer	PIWIL3	Tumor suppressor	progression-free and overall survival, neural invasion	[244]
	Pancreatic cancer	PIWIL4	Tumor suppressor	progression-free and overall survival, neural invasion, T3-stage	[244]
	Pancreatic cancer	piR-017061	Tumor suppressor	poor overall survival	[91]
	Hepatocellular carcinoma	PIWIL1	Carcinogen	NA	[118]
Urinary system cancers	Renal cell carcinoma	piR-57,125	Tumor suppressor	tumor metastasis, poor overall survival	[104]
	Renal cell carcinoma	piR-31,115	Carcinogen	NA	[172]
	Clear cell renal cell cancer	piR-30,924 and piR-38,756	Carcinogen	tumor metastasis, poor overall survival	[245]
	Prostate cancer	piR-001773 and piR-017184	Carcinogen	shorter overall survival	[101]
	Prostate cancer	piR-31,470	Carcinogen	NA	[139]
	Prostate cancer	piR-19,166	Tumor suppressor	tumor metastasis and lymph node metastasis	[105]
	Prostate cancer	PIWIL2	Carcinogen	lymph node metastasis, and advanced TNM stage	[108]
	Bladder Cancer	piR-5936	Carcinogen	increased risk, shorter survival	[246]
	Bladder Cancer	piR-60,152	Tumor suppressor	NA	[247]
Reproductive System cancers	Breast cancers	piR-823	Carcinogen	NA	[137]
	Breast cancers	piR-651	Carcinogen	NA	[144]
	Breast cancers	piR-36,712	Tumor suppressor	longer progression-free survival, lymphatic metastasis,	[92]
	Breast cancers	piRNA-17,560	Carcinogen	Increase chemotherapy resistance	[112]
	Cervical cancer	PIWIL2	Carcinogen	pathological grade, drug resistance	[248] [249]
	Cervical cancer	piRNA-14,633	Carcinogen	advanced TNM stage and increased tumor size	[141]
	Ovarian cancer	piR-52,207 and piR-33,733	Carcinogen	NA	[250]
	Endometrial cancer	PIWIL1	Carcinogen	promote endometrial cancer cell stemness	[113] [137] [138]
Hematologic Cancers	Acute leukemia	hsa_piR_011186	Carcinogen	NA	[143]
	DLBCL	piRNA-30,473	Carcinogen	overall survival and poor prognosis	[140]
	Multiple Myeloma	piRNA-823	Carcinogen	ISS III, shorter overall survival.	[129] [111]
	Multiple Myeloma	PIWIL1	Carcinogen	ISS II and ISS III	[251]
	Myeloma	piR-004800	Carcinogen	ISS III and MM progression	[93]
	Classical Hodgkin lymphoma	piR-651	Tumor suppressor	disease-free survival and overall survival	[252]
Respiratory cancers	Lung Adenocarcinoma	piR-hsa-211,106	Tumor suppressor	NA	[116]
	Non-small cell lung cancer	piR-651	Carcinogen	distal metastasis and recurrence, inversely correlated with overall survival	[97] [98]
	Lung cancer	piR-34,871 and piR-52,200	Carcinogen	NA	[253]
	Lung Adenocarcinoma	piR-57,125	Tumor suppressor	distant metastasis	[254]
	Lung cancer	piR-55,490	Tumor suppressor	longer survival time	[103]
Nervous system cancers	Glioblastoma	piR-9491 and piR-12,488, piR-23,231	Tumor suppressor	Overall survival	[255]
	Glioblastoma	piRNA-8041	Tumor suppressor	NA	[94]
	Glioblastoma	piR-30,188/PIWIL3	Tumor suppressor	negatively correlated with the pathological grade	[96]
	Glioblastoma	PIWIL1	Carcinogen	larger tumor diameter and advanced tumor grading	[95] [256]
	Glioblastoma	PIWIL1/piRNA-DQ593109	Carcinogen	drug resistance and poor prognosis	[257]
	Neuroblastoma	piR-39,980	Carcinogen	clinical drug resistance	[258]
Other kind of cancers	Fibrosarcoma	piR-39,980	Tumor suppressor	promotes sensitivity of fibrosarcoma to DOX	[119]
	Fibrosarcoma	PIWIL4	Tumor suppressor	low expression with poor disease-free survival, disease-specific survival, and progression-free survival	[120]
	Osteosarcoma	piR-39,980	Carcinogen	invasion and metastasis	[106]
	papillary thyroid carcinoma	piR-13,643 and piR-21,238	Carcinogen	tumor stage	[259]
Nervous system disease	Alzheimer’s disease	piR-34,393 and piR-38,240	Promoter	causes neurodegeneration in AD	[260]
	Sporadic Amyotrophic Lateral Sclerosis	PIWIL1 and PIWIL4	Protective factor	NA	[261]
Reproductive system diseases	Preeclampsia	PIWIL1	Protective factor	earlier gestational weeks, lower birth weight, lower Apgar score, and higher incidence of postpartum hemorrhage.	[262]
	Adenomyotic lesions	PIWIL2	Promoter	NA	[263]
	Infertility	PIWIL1 mutation	Harmful factor	impair sperm quantity and quality	[264]
Cardiovascular and circulatory diseases	Pulmonary arterial hypertension	piRNA-63,076	Harmful factor	increase pulmonary vascular resistance	[136]
	Cardiac hypertrophy	piR-141,981	Harmful factor	promote cardiomyocyte hypertrophy	[142]
	Myocardial Infarction	piR_2106027	Harmful factor	associated with myocardial injury	[265]
	Trypanosoma cruzi infection in cardiac myocytes	piR46, piR573 and piR587	Harmful factor	promote myocardial cell fibrosis	[266]
	Lung fibrosis	PIWIL2	Protective factor	inhibit radiation-induced pulmonary fibrosis	[117]
	Cerebral ischemic injury	PIWIL2	Harmful factor	downregulation of PIWIL2 contributes to the neuroprotection	[139]
Other kind of Disease	HIV-1 Latency	PIWIL4	Protective factor	inhibit HIV-1 pro-viral DNA expression and establishes and maintains HIV-1 latency in CD4 + T lymphocytes	[122]

### Digestive system cancers

Most new cases of oral cancer diagnosed each year worldwide are squamous-cell cancers leading to painful deaths [267, 268]. The expression of PIWIL2 is significantly increased in all oral cancer cell lines. Patients with high PIWIL2 have more lymph node metastasis, poor histological type, clinical stage, and shorter survival [231, 232]. piR-1037 promotes chemotherapy resistance of OSCC cells to cisplatin (CDDP) by promoting invasion and metastasis via EMT, leading to poor prognosis [114].

Esophageal cancer is the sixth leading cause of cancer death worldwide, with esophageal squamous cell carcinoma (ESCC) accounting for about 90% of all esophageal cancers [269–272]. Su et al. reported that piR-823, highly expressed in ESCC, was positively correlated with pathological features such as lymph node metastasis and tumor size [130]. PIWIL2 is highly expressed in ESCC with a positive correlation with pathological T-stage and a negative correlation with long-term survival rate. In vitro experiments confirmed that it aided malignancy by inhibiting apoptosis [99].

Gastric cancer (GC) is the third leading cause of cancer death worldwide [273, 274]. piR-823 levels in gastric cancer tissues were significantly lower than in para-cancerous tissues. Furthermore, elevated piR-823 levels significantly inhibited tumor growth in vivo in a dose-dependent manner. However, the specific mechanism has not been further studied [132]. In addition, piRNA-1245 expression positively correlated with tumor size and TNM stage and inversely with overall survival in GC patients [233]. Moreover, the upregulated PIWIL1 in gastric cancer samples significantly correlated with tumor node metastasis but inversely with the degree of differentiation. High PIWIL1 mRNA expression is associated with poor prognosis, frequently seen in patients with poor differentiation and high TNM stage [234].

The development of clinical research and molecular targets have increased the treatment options for patients with advanced colorectal cancer [275–277]. However, colorectal cancer (CRC) is still the fourth deadliest cancer globally [278–280]. The highly expressed piR-020619 and piR-020450 act as oncogenes in CRC. The expression levels of these piRNAs in serum closely correlated to tumor size and early stage. The piR-02061 and piR-02450 detected in serum samples of patients after surgery were significantly lower than those before surgery [235]. Mai et al. demonstrated that the serum level of piR-54,265 in CRC patients is significantly higher than in the control groups, suggesting that its expression significantly increases in patients with advanced and metastatic colorectal cancer. Moreover, the overall survival due to its high expression was significantly shorter than those with low piR-54,655 levels [107]. In addition, the expression of piR-54,265 in stage I CRC and high-grade intraepithelial neoplasia (HIN) were significantly higher than that in the control group, suggesting that abnormally high piR-54,265 may be an early event in CRC development [236]. piR-24,000 is highly expressed in CRC, positively correlated with tumor stage and poor differentiation. In addition, distant metastases of tumors had a significant correlation with high piR-24,000 expression [237]. Compared with the healthy control group, piR-823 in tumor tissues correlated with tumor stage and differentiation, especially in poorly differentiated tumor tissues and stages III and IV. In serum, piR-823 content correlated with the advanced clinical stages (III and IV), poor differentiation, and lymph node metastasis of CRC [90, 115, 238]. Overexpression of PIWIL1 inhibits the apoptosis of CRC cells, promotes angiogenesis, and increases tumor volume and weight in nude mice, thus promoting the malignant progression of CRC [239, 240].

Pancreatic ductal adenocarcinoma (PDAC) is the most common form of pancreatic cancer, with a doubled global burden in the past 25 years [281–283]. About 50% of new diagnoses are metastatic, with a mean survival of less than one year [284, 285]. piR-017061 was reported to be inversely associated with long-term survival in PDAC [91]. High expression of PIWIL2 protein in pancreatic cancer patients is statistically significantly correlated with PFS and overall survival than those with low expression. Low PIWIL2 expression is associated with higher T stage, significantly correlating with other pathological features, such as vascular and perineural invasion, tumor stage, or lymph node involvement, leading to tumor aggressiveness [243]. Li et al. demonstrated how high PIWIL1 expression enhances the invasiveness of pancreatic cancer cells, promoting cancer progression [109]. Patients with low PIWIL3 or PIWIL4 expression showed shorter PFS and overall survival than those with high expression of both proteins. In addition, low PIWIL3 levels were significantly associated with neuronal invasion. Low PIWIL4 expression correlates with a higher risk of postoperative recurrence. They were significantly related to patients with T3-stage [244].

Pancreatic fibrosis is a prominent pathological feature of pancreatic cancer caused by pancreatic stellate cells (PSCs) [286–288]. PIWIL1 group significantly reduced the expression levels of type I and III collagen and α-smooth muscle actin, and then inhibited PSCs’ invasion and migration ability. This observation indicates PIWIL1 to be a potential therapeutic target for pancreatic fibrosis to prevent pancreatic cancer indirectly [289].

Intrahepatic cholangiocarcinoma (ICC) is a malignant hepatobiliary tumor from the bile duct with difficult diagnoses, poor prognosis, and a high mortality rate [290–292]. PIWIL4 upregulation was identified as a critical biomarker in this cancer, proportional to patient survival [241]. In addition, the levels of piR-10,506,469 in plasma exosomes of CCA and GBC patients were significantly increased. In contrast, the levels of piR-10,506,469 and piR-20,548,188 in postoperative patients were significantly decreased, as detected by comparing the differential expression of extracellular piRNA in patient plasma before and after surgery. This suggests that the expression levels of piR-10,506,469 and piR-20,548,188 negatively correlated with the patient prognosis [242].

Hepatocellular carcinoma (HCC) is the third leading cause of cancer-related death worldwide [129, 293]. The expression of PIWIL1 was significantly upregulated in HCC tissues, accelerating the proliferation of HCC cells and inducing colony formation in vitro, increasing the volume of HCC grafts in vivo [118].

### Respiratory cancers

Lung cancer cases and deaths are on the rise globally, causing the most cancer deaths in the population [294, 295]. Of all types of lung cancer, non-small cell lung cancer (NSCLC) is the most common subtype [296–298].

piR-651 is highly expressed in NSCLC, positively correlated with distant metastasis and disease recurrence, and negatively associated with overall survival [97, 98]. As a tumor suppressor, patients with high expression of piR-55,490 have more prolonged survival and better prognoses [103]. piR-hsa-211,106 inhibits proliferation, promotes apoptosis, and attenuates the invasion ability of Lung adenocarcinoma (LUAD) cells. In addition, it can also increase the sensitivity of cells to Cisplatin to improve the prognosis of patients with drug resistance [116]. piR-57,125 is mainly related to the invasiveness of lung adenocarcinomas (LAC). piR-57,125 expression is significantly down-regulated in metastatic LACs compared with non-metastatic LACs [299]. Moreover, the tumor promoter RASSF1C upregulates the oncogenic factors piR-34,871 and piR-52,200 via the RASSF1C-PIWIL1-PIRNA axis to promote LC stem cell proliferation, colony formation, and EMT [254]. However, the clinical impact of piR-34,871 and piR-52,200 on lung cancer has not been reported.

### Urinary system cancers

Clear cell renal carcinoma (ccRCC) is the sixth most common cancer in the world, often with a poor prognosis [253, 300–302]. Moreover, ccRCC is highly resistant to chemotherapy and radiotherapy [303, 304]. piR-57,125 was reported to be down-regulated in ccRCC, especially in metastatic tumors, implying a plausible role of piR-57,125 as a tumor suppressor by inhibiting tumor metastasis [104]. In contrast, piR-30,924 and piR-38,756 are highly expressed in primary metastatic tumors and have decreased expression in non-metastatic tumors compared to normal tissue. Their high expression is also significantly associated with tumor recurrence and poor overall survival [305].

Prostate cancer (PCa) is the second most common cancer and the fifth leading cause of cancer-related death in men [245, 306]. PIWIL2 has a high expression in PCa, which positively correlates with Gleason score, lymph node metastasis, and advanced TNM stage (T3-T4) [108]. piR-19,166 is down-regulated in PCa and closely related to organ and lymph node metastasis [105]. Moreover, upregulation of piR-001773/piR-017184 leads to shorter overall survival [101].

Bladder cancer (BLCA) is a common malignancy with high morbidity and mortality [307–309]. Sabo et al. used next-generation sequencing to piR-hsa-5936 upregulation in plasma EVs to be a risk factor for bladder cancer [310]. As a newly discovered tumor suppressor in bladder cancer, piR-60,152 has not been proven to have clinical significance. However, in vivo experiments have shown that its overexpression inhibits the proliferation and colony formation of bladder cancer cells and promotes apoptosis [246].

### Reproductive system cancers

Breast cancer was the most common cancer and the leading cause of cancer-related death among women worldwide [247, 311–313]. piR-36,712 is significantly down-regulated in breast cancer. A slightly higher expression of piR-36,712 significantly affects progression-free survival, while its low expression is closely related to axillary lymph node metastasis [92]. In BC patients receiving chemotherapy, the exosome piR-17,560 enhances resistance of BC cells, developing drug resistance and leading to poor prognosis [112].

More than half a million women are diagnosed with cervical cancer each year, resulting in 300,000 deaths, with metastatic and recurrent patients having the worst overall prognosis [314–316]. Xie et al. reported that piRNA-14,633 is highly expressed in cervical cancer and is closely related to the advanced TNM stage and increased tumor size [141]. In recent years, PIWIL2 has been proved to promote the reprogramming of normal cervical stem cells to become cervical cancer cells. PIWIL2 was significantly increased in CIN2/3 and weakly expressed in CIN1, indicating the tissue specificity of PIWIL2 [248, 317].

Ovarian cancer (OCa) is a fatal gynecological cancer, the leading cause of death in women over the past few years [249, 318]. The most common type is epithelial ovarian cancer (EOCa), with endometrioid ovarian cancer (ENOCa) and serous ovarian cancer (SOCa) being the most lethal subtypes [319–321]. piR-52,207 upregulates ENOCa and promotes cell proliferation, migration, and tumorigenesis. At the same time, piR-52,207 and piR-33,733 are upregulated in SOCa, leading to apoptosis inhibition in the cells [322].

Endometrial cancer (EC) is one of the most common reproductive system malignancies [250, 323]. Clinically, most endometrial cancers are type I, also known as estrogen-dependent endometrioid adenocarcinoma [324, 325]. PIWIL1 acts as an oncogene in endometrial cancers, inducing EMT and conferring stemness characteristics on EC cells, promoting cancer growth [113]. In addition, PIWIL1 inhibits its downstream target PTEN and can act as a downstream target of ERα and participate in E2-stimulated endometrial carcinogenesis [137, 138].

### Hematologic cancers

Hematologic malignancies have a highly variable prognosis and continuous post-treatment relapse [326–328]. Among them, leukemias is the most common pediatric cancer [329]. Hsa_piR_011186 significantly pushes the cell cycle into the S phase and inhibits apoptosis, thereby promoting the occurrence and development of acute leukemia [133].

Diffuse large B-cell lymphoma (DLBCL) is the most common subtype of lymphoid malignancies [330]. And current treatment methods for advanced patients still keep 40% of patients uncured [331, 332]. It is reported that high expression of piR-30,473 is associated with poor long-term prognosis of DLBCL patients, with the overall survival being inversely proportional to the expression level [140].

Multiple myeloma (MM) is an incurable malignant tumor derived from B cells [333]. The fundamental difficulty of its treatment is bolstered by the unlimited self-renewal ability of a few cancer stem cells (CSCs) [334]. piR-004800 is highly expressed in the bone marrow supernatant exosomes of MM patients and significantly increases in ISS III patients, suggesting its upregulation be correlated with MM progression [93]. High expression of piR-823 was associated with a higher ISS stage and shorter survival time. Granulocytic MDSCs (G-MDSCs) trigger piRNA-823 expression, increasing the tumorigenic potential of MM cells [111, 129]. PIWIL1 expression was significantly high in ISSII and III stage MM patients than in stage I, suggesting it to be associated with an advanced stage of the disease. In addition, higher PIWIL1 levels in refractory MM patients than in newly diagnosed suggested a gradual increase in PIWIL1 expression from freshly diagnosed to relapsed/refractory MM patients [26].

Classical Hodgkin lymphoma (cHL) accounts for a small percentage of all lymphomas [251, 335]. The low expression of piR-651, a tumor suppressor, is associated with shorter disease-free and overall survival [336].

### Nervous system cancers

Glioblastoma (GBM), the most lethal form of glioma in adults, is the most common primary brain tumor [252, 337, 338]. Overexpression of piR-8041 inhibited long-term colony formation and glioma cell proliferation. In addition, tumors induced in mouse brains showed that overexpression of piR-8041 significantly inhibited tumor volume. The effectiveness of piR-8041 requires maintained periodic therapy, which in clinical practice largely depends on the efficacy of the vector in crossing the blood-brain barrier (BBB) [94]. Bartos et al. found that low expression of piR-23,231 was significantly associated with decreased survival. In addition, piR-hsa-9491 and piR-has-12,488 significantly reduced the colony formation ability of GBM cells in vitro [339]. The high PIWIL1 expression in GBM correlates with larger tumor diameter and higher tumor grade, promoting the malignant proliferation of glioma stem cells (GSCs). It also reduces the effect of drug therapy by reducing the blood-tumor barrier (BTB) [95, 255, 340]. The tumor-suppressor PIWIL3 has a low expression in glioma and a negative correlation with the pathological grade of the disease. In contrast, overexpression of PIWIL3 significantly inhibited the progression of glioma [96].

Neuroblastoma, cancer originating in the neural crest, is the most common extracranial solid tumor in children [256, 257]. Although the lesion may spontaneously regress in some cases, it can sometimes lead to severe attacks [341, 342]. As a cancer-promoting factor, piR-39,980 overexpression significantly improves the survival of NB cells, promoting invasion and metastasis. Moreover, the higher expression of piR-39,980 reduced the sensitivity of NB cells to doxorubicin. It may contribute to the development of chemotherapy resistance, which is closely related to NB’s current clinical drug resistance [343].

### Other kinds of cancers

Fibrosarcoma represents only a tiny percentage of all soft tissue sarcomas [344]. Currently, the treatment of metastatic fibrosarcoma mainly relies on chemotherapy [258, 345]. piR-39,980 is downregulated in fibrosarcoma cells, causing doxorubicin resistance. Conversely, high expression of piR-39,980 attenuates doxorubicin resistance [119]. Only 5% of fibrosarcoma patients show high PIWIL4 expression, which positively correlates with survival, disease-free survival, disease-specific survival, and progression-free survival [120].

Osteosarcoma (OS) is the most common malignancy of the skeletal system with high invasion and metastasis ability [346, 347]. The prognosis of metastatic or recurrent osteosarcoma is still unsatisfactory [348, 349]. The high expression of piR-39,980 correlates to osteosarcoma’s high invasion and metastasis. Silencing the tumor suppressor SERPINB1 and MMP-2 induction induces the invasion and metastasis ability of osteosarcoma cells [106]. Chang et al. showed the high expression of piR-13,643 and piR-21,238 in thyroid cancer, associated with clinical stages [350].

### Association of piRNAs/PIWI proteins with clinical characterization in diverse non-cancer diseases

#### Nervous system diseases

Alzheimer’s disease (AD), the most common form of dementia, is characterized by progressive memory loss and other cognitive abilities [259, 351]. Cytochrome C (CYCS), released from the mitochondrial membrane, induces the piR-38,240- and piR-34,393-mediated regulation of apoptosis by triggering the activation of caspase cascades. In addition, piR-38,240 reduces the expression of another protective factor, KPNA6, which regulates NRF2-dependent antioxidant responses. Together, these two mechanisms contribute to nerve cell damage in AD [352].

Amyotrophic lateral sclerosis (ALS) is a fatal neuromuscular disease and results in muscle dysfunction [260, 353, 354]. In Sporadic Amyotrophic Lateral Sclerosis (sALS), the expressions of the protective factors PIWIL1 and PIWIL4 are significantly downregulated. In the absence of this neurodegenerative disease, PIWIL1 is highly expressed in anterior horn cells. In contrast, the lumbar spine of sALS patients shows a drastic reduction in the number of anterior horn cells, which are PIWIL1-negative. Previous research has implicated the crucial role of PIWIL1 in neuronal development and protection in the migration of microtubules [355]. This observation suggested that PIWIL1 expression change can act as a compensatory mechanism for axonal dysfunction [356].

#### Cardiovascular and circulatory systems diseases

*T.cruzi*, which can penetrate the interstitial vascular system and eventually invade cardiomyocytes, causes heart disease and results in myocardial damage and conduction abnormalities [261, 357]. *T. cruzi* has a highly differential expression of the fibrosis gene, such as metalloproteinase families MMP2, MMP9 and MMP12, as well as abnormal expression of TGF-β, which ultimately causes cardiac fibroblasts to differentiate into myofibroblasts [358–360]. The expression of some novel piRNAs is significantly induced at the early stage of the infection. Studies have identified a novel piRNA (npiR46) that binds to NFATC2 and FOS, and npiR573, and npiR587 bind to TGFβ1, promoting fibrosis. These piRNAs are important biomarkers for the early stage of *T.cruzi* infection [361].

Chronic hypertrophy and its associated myocardial remodeling significantly contribute to cardiac dysfunction, leading to severe heart failure and death [266, 362]. Overexpression of piR-141,981 enhanced Ang II-induced cardiomyocyte hypertrophy and increased Ang II-induced ANP expression, encoding the atrial natriuretic peptide. In addition, it inhibits the activation of RNA epigenetic modules of anti-hypertrophic molecules such as GSK3β and upregulates PARP10 as a pro-hypertrophic factor [142]. Rajan et al. found piR_2106027 to be significantly upregulated in patients with myocardial infarction and correlated with troponin I levels, indicating piR_2106027 expression to be associated with the degree of myocardial injury [363]. This has the potential to become a new clinical biomarker for patients with myocardial infarction.

Abnormal proliferation of pulmonary artery smooth muscle cells (PASMCs) and hypoxia-induced abnormal secretion of extracellular matrix proteins increase pulmonary vascular resistance and pulmonary hypertension (PH) [265, 364, 365]. A higher expression of piR-63,076 was seen in the medial region of hypoxic pulmonary arteries but not in normal lung tissues. piR-63,076 knockdown attenuated the development of pulmonary hypertension by inhibiting the proliferation of PASMCs, implicating its potential role as a biomarker and a therapeutic target for pulmonary hypertension [136].

Radiotherapy is considered to be an effective treatment for thoracic cancers such as that of the lung and breast [297, 366–368]. However, a significant complication of radiotherapy, radiation-induced pulmonary fibrosis (RILF), not only limits radiotherapy efficacy but also seriously affects patients’ quality of life [369, 370]. Upregulation of Nrf2 abolishes RILF in vitro and in vivo. Nrf2 activates its downstream target PIWIL2 to increase its expression. More importantly, the knockdown of PIWIL2 significantly reverses the protective effect of Nrf2. In conclusion, upregulation of Nrf2 and PIWIL2 might potentially have a protective role in RILF reversal [117].

Stroke is the second leading cause of death globally, with ischemic strokes accounting for at least 80% of all cases [371]. In this condition, there is an irreversible loss of brain cells due to ischemia and hypoxia [372–374]. Downregulated PIWIL2 contributes to neuroprotection against transient global cerebral ischemia (tGCI) in CA1 neurons induced by hypoxic postconditioning (HPC). HPC entirely prevents the tGCI-induced increase in PIWIL2 mRNA and enhanced hippocampal dendritic spine plasticity, improving memory function after tGCI [139].

#### Reproductive system diseases

Preeclampsia is an idiopathic pregnancy disorder that occurs after 20 weeks of gestation, severely affecting the health of the pregnant woman and the fetus [375–377]. PIWIL1 is highly expressed in trophoblasts, promoting their proliferation and invasion of trophoblasts and inhibiting apoptosis. PIWIL1 is reduced in preeclampsia, inhibits the proliferation of trophoblast cells, and promotes apoptosis of placental trophoblast cells, leading to shallow placenta accreta and the development of preeclampsia [378].

Adenomyosis is a benign reproductive disease characterized by the invasion of endometrial tissue into the myometrium [379]. PIWIL2 is highly expressed in adenomyosis and associated with disease progression. It is hypothesized that PIWIL2 may be involved in endometrial cell proliferation, critically mediating the disease pathogenesis; however, the specific underlying mechanisms need further study [262]. PIWIL1 is a critical E3 ligase, chelating histone ubiquitination in the cytoplasm of early sperm cells. The level of its mutants is related to the degree of developmental defects in sperms. Degradation of PIWIL1 by APC/C in advanced sperm cells releases RNF8 into the nucleus to mediate subsequent histone-to-protamine conversion. In contrast, PIWIL1 mutations block the RNF8 nuclear translocation and RNF8-mediated histone to induce protamine conversion in advanced sperm cells, resulting in impaired sperm count and quality [380].

#### Other kinds of diseases

PIWIL4 has been implicated in playing a significant role in suppressing HIV-1 transcription by recruiting various repressors. It promotes the latent state of HIV-1-infected cells. Endogenous PIWIL4 inhibits HIV-1 pro-viral DNA expression and maintains HIV-1 latency in CD4 + T lymphocytes. PIWIL4 knockdown enhances HIV-1 transcription and reverses HIV-1 latency in Jurkat T cells and primary CD4 + T lymphocytes. Mechanically, PIWIL4 depletion reactivates HIV-1 replication in latently infected primary CD4 + T lymphocytes [122]. This may provide new ideas for the treatment of AIDS, by promoting the expression of PIWIL4 to enforce the maintenance of HIV virus in the latent state.

#### Clinical application of piRNAs

Early detection and rapid evaluation of the progress of an acute cardiovascular disease or highly fatal cancer provide a valuable reference for future treatment [263, 264, 381, 382]. The expression of piRNA, a newly discovered small ncRNA, shows a statistically significant correlation between serum or body fluids and disease [383–385]. In this section, we summarize the potential applications of piRNA in clinical applications (Tables 4 and 5).

**Table 4:** Prospects for the clinical applications of piRNAs and PIWI proteins as biomarkers

Source	piRNAs	Cancer type	Role in cancers	Clinical value	Ref.
Tissue	piR-823	Esophageal cell squamous carcinoma	Carcinogen	Sensitivity and specificity	[130]
Tissue	piR-24,000	Colorectal cancer	Carcinogen	Sensitivity and specificity	[237]
Tissue	piR-30,924 and piR-38,756	Clear cell Renal cell carcinomas	Carcinogen	Closely related to tumor metastasis	[245]
Tissue	piR-57,125	Clear cell Renal cell carcinomas	Tumor suppressor	Closely related to tumor metastasis	[245]
Tissue	piR-9491	Glioblastoma	Tumor suppressor	Closely related to shorter survival	[255]
Tissue	piR-13,643 and piR-21,238	Papillary thyroid carcinoma	Carcinogen	Distinguish malignant from benign nodules	[259]
Sera	piR-020619	Colorectal cancer	Carcinogen	Sensitivity and specificity	[235]
Sera	piR-020450	Colorectal cancer	Carcinogen	Sensitivity and specificity	[235]
Sera	piR-54,265	Colorectal cancer	Carcinogen	stable for detection and Quantification, high sensitivity and specificity	[236]
Sera	piR-651	Classical Hodgkin lymphoma	Tumor suppressor	Closely related to disease-free survival and overall survival	[252]
Tissue and Sera	piR-823	Colorectal cancer	Carcinogen	Distinguish malignant from benign nodules, and high correlation between tissue and serum	[238]
Gastric juice and tissue	piR-1245	Gastric Cancer	Carcinogen	Sensitivity and specificity	[233]
Plasma Evs and Urine	piR-5936	Bladder Cancer	Carcinogen	Non-invasive biomarkers	[246]

**Table 5:** Prospects for the clinical applications of piRNAs and PIWI proteins in drug resistance

piRNA/PIWI proteins	Diseases	Role in cancers	Function	Functional mechanism	Ref.
PIWIL1	Multiple Myeloma	Carcinogen	Increase MM cell survival and promoted cell proliferation	Anti-autophagy and anti-mitophagy	[251]
PIWIL1/piRNA-DQ593109	Glioblastoma	Carcinogen	Decrease BTB Permeability	Decrease the concentration of chemotherapy drugs inside the tumor	[257]
piR-39,980	Neuroblastoma	Carcinogen	Induce the cancer cell growth, enhance metastasis, and inhibit the cellular senescence	Decrease the sensitivity of neuroblastoma to doxorubicin	[258]
piR-39,980	Fibrosarcoma	Tumor suppressor	Induce apoptosis and anti-proliferation effects	Increase sensitivity of fibrosarcoma to DOX	[119]
piR-hsa-211,106	Lung Adenocarcinoma	Tumor suppressor	Inhibit the proliferation and migration and promote apoptosis	Enhance the Chemotherapy Sensitivity of Cisplatin	[116]
PIWIL2	Cervical cancer	Carcinogen	Maintain the chemotherapy resistance of tumor initiation cells	Decrease the sensitivity of cisplatin in cervical cancer	[248]
piR-1037	OSCC	Carcinogen	Inhibit apoptosis	Decrease the sensitivity of OSCC to cisplatin	[114]

#### piRNAs as diagnostic and prognostic biomarkers in cancer

**Fig. 4:**
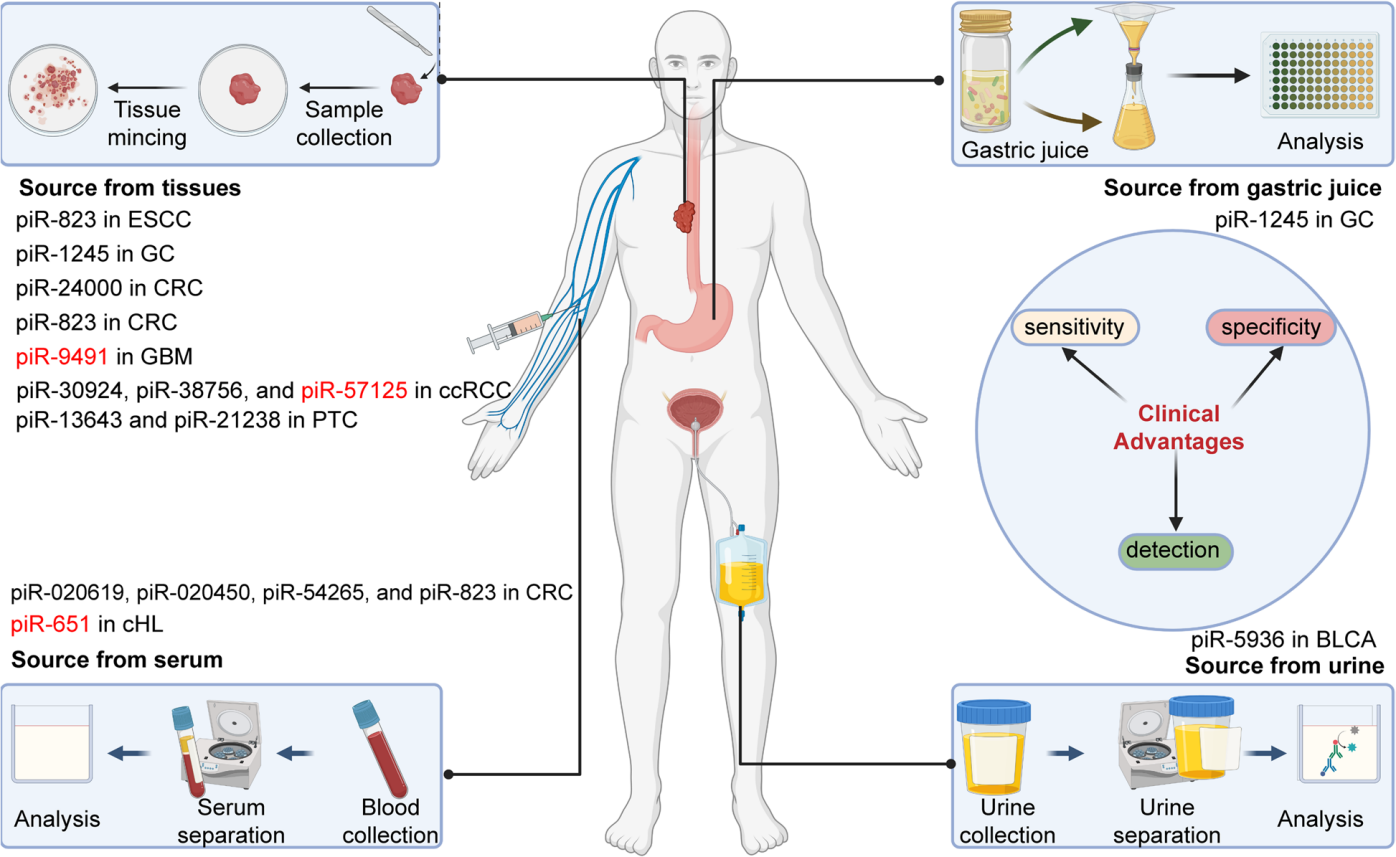
Potential value of piRNAs/PIWI proteins as clinical markers. piRNAs/PIWI proteins are mainly detected in tissues, serum, gastric juice and urine as biomarkers. As a biomarker, it also needs to meet the characteristics of high sensitivity, specificity, and convenient detection. Among them, the red targets are tumor suppressor factors, and their expression is positively proportional to the prognosis of patients. The black target is the pro-cancer factor, and the higher its expression, the worse the prognosis of the patient

piRNAs/PIWI proteins are great potential clinical markers possessing the following characteristics: sensitivity, specificity, expression stability, and broad applicability [386]. According to their various sources, they can be roughly divided into tissue-derived, serum-derived, and body-fluid-derived ones (Fig. 4).

Tissue-derived biomarkers are often stably expressed with high specificity [387]. Su et al. further tested the diagnostic value of piR-823 in ESCC using ROC plots, which showed piR-823 to be a valuable biomarker with a sensitivity of 62.96% and a specificity of 76.93%, accurately distinguishing ESCC patients from normal individuals [130]. piR-24,000 in CRC tissues could significantly distinguish patients from normal subjects. In the late group (stages III and IV), the AUC value is 0.8405, while in the early group (stages I and II), the AUC value was slightly lower at 0.7796 [237]. piR-57,125, piR-30,924, and piR-38,756 are potential prognostic biomarkers of ccRCC because the high expression of piR-30,924 and piR-38,756 and low expression of piR-57,125 significantly correlates with the metastatic tumors and bone metastasis. The prognostic significance of piR-38,756 was particularly indicative in nonmetastatic patients [305]. piR-9491 showed the best specificity and sensitivity for identifying GBM samples from non-tumor samples. Its low expression significantly correlated with shorter survival [339]. The piRNA sequencing data of papillary thyroid cancer revealed two upregulated piRNAs, piR-13,643 and piR-21,238, showing better specificity than the current biomarker HBME1 in distinguishing benign and malignant nodules and were closely related to prognosis [350].

Serum-derived biomarkers often show high sensitivity and prognostic associations, reflecting the status of patients on time [388–390]. In addition, they can also reflect the status of patients in a timely manner. Most people with early-stage colorectal cancer have few or no symptoms [391, 392]. The traditional serum tumor markers CEA and CA19-9 are still unsuitable for colorectal cancer screening due to their low sensitivity [393, 394]. The AUC of piR-020619 and piR-020450 were 0.883 and 0.913, respectively. The sensitivity and specificity of these piRNAs were higher than the currently used biomarkers CEA and CA199. And the serum levels of piR-020619 and piR-020450 of colorectal cancer patients after surgery also decreased. In addition, for early-stage and small CRC tumors less than 3 cm, the sensitivity of the piRNA group was much higher than that of CEA and CA19-9, and the specificity of the piRNA group was 90.94%, similar to that of CEA and CA19-9. This phenomenon is best demonstrated by the piRNA panel’s ability to distinguish CEA and CA19-9-negative CRC patients from normal controls [235]. Stability is a critical characteristic of biomarkers, playing a crucial role in clinical convenience [395, 396]. Circulating piR-54,265 is stable in CRC serum to be readily and reliably measured. In addition, Mai et al. also verified the sensitivity and specificity of piR-54,265 to be significantly higher in the serum of CRC patients than in all other control groups [107]. Mai et al. further investigated the specificity of piR-54,265 in CRC precancerous lesions, which revealed that the expression level of piR-54,265 in patients with HIN or stage I CRC was significantly higher than that in controls, implying that abnormal overexpression of piR-54,265 may be an early event in CRC development. The serum piR-54,265 level in postoperative CRC patients showed a dynamic decline. In contrast, serum indicators also showed a synchronous rise in recurrent patients, and its dynamic changes were more accurate than the current CEA, CA19-9, and CA125, indicating that serum piR-54,265 are potential new effective indicators of CRC recurrence [236]. Compared with the healthy control group, the expression of piR-651 in the serum of the CHL group was low. After the complete response, the expression of piR-651 was upregulated again and reached almost the same standard as that of the healthy control group, indicating that piR-651 has the potential to be a prognostic marker [336]. Specifically, piR-823 was differentially expressed in tissues and serum compared with normal tissues. Tissue expression of piR-823 was significantly associated with poorly differentiated tumor tissue and stage III and IV. And serum piR-823 content was associated with the advanced clinical stage (III and IV), poor differentiation, and lymph node metastasis. Correlation analysis demonstrated that the increase of piR-823 in tissues correlated with its increase in serum, promising to replace invasive colonoscopy with noninvasive blood sampling [238].

Finally, body fluid-derived biomarkers, such as piRNAs from gastric juice and urine, hold the potential to improve the convenience of detection. They can significantly avoid the invasive examination caused by routine biomarker collection. piR-1245 is a highly expressed oncogenic factor in gastric cancer, and its high expression positively correlates with overall poor survival, suggesting poor prognosis. Statistical results show that the sensitivity and specificity of piR-1245 were higher than those of CEA and CA724, commonly used gastric cancer biomarkers [233]. Therefore, it can potentially replace the currently known gastric cancer markers and help early-stage detection of gastric cancer in patients. Cystoscopy and urine cytology are the gold standards for diagnosing BLCA, but with limited convenience. Therefore, it is necessary to find highly sensitive and specific body fluid markers to facilitate high-risk screening groups. Compared with the control group, the expression of piR-hsa-5936 in BLCA cases showed an upward trend, resulting in an increased risk of BLCA and a shorter survival period. However, only plasma EV showed its differential expression but not urine [310].

#### piRNAs and cancer therapy resistance

**Fig. 5:**
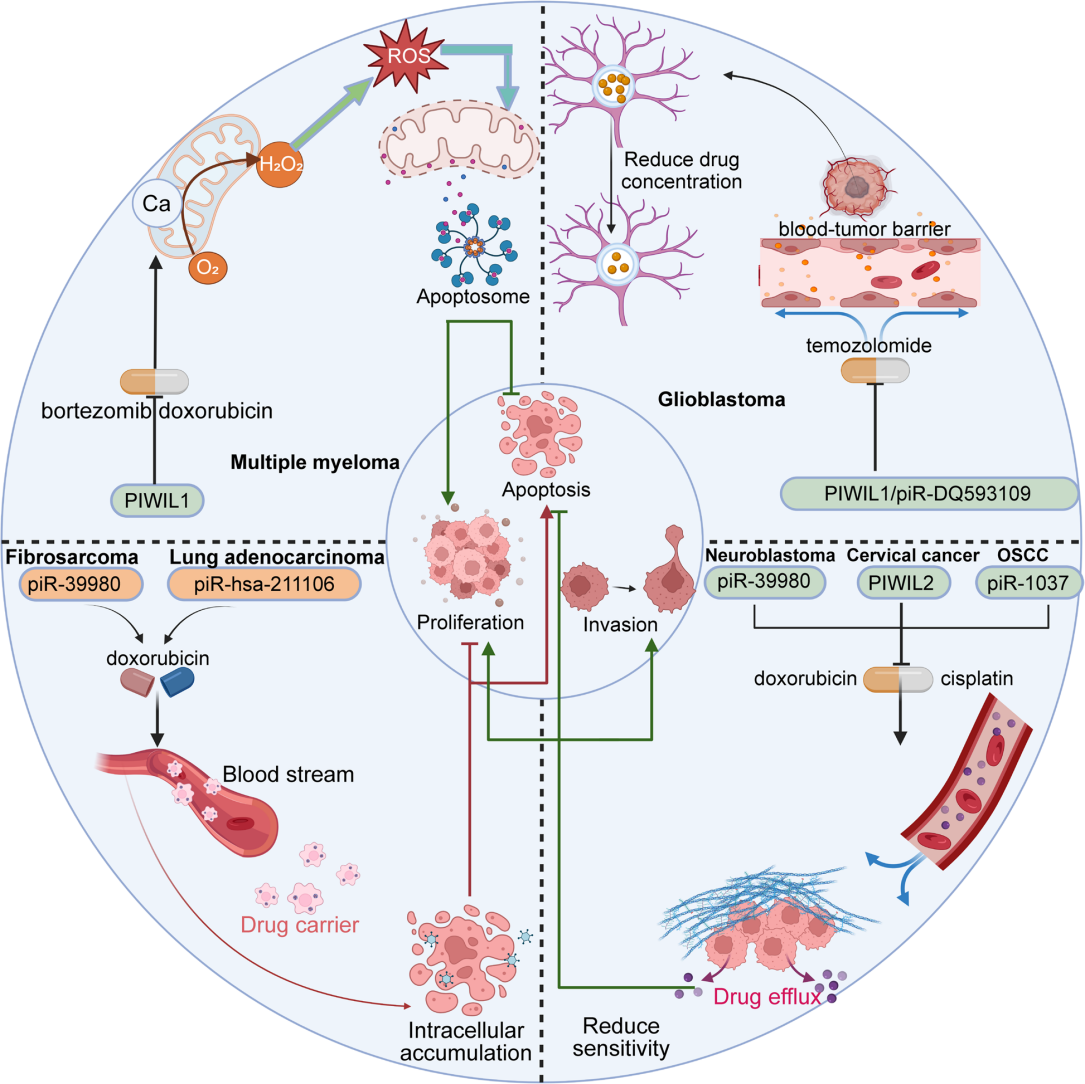
The green targets in the figure are piRNAs/PIWI proteins that increase chemotherapy resistance. PIWIL1 inhibits the pro-apoptotic effect of chemotherapeutic drugs on MM cells by inhibiting mitochondrial apoptosis, thereby promoting proliferation and inhibiting apoptosis. PIWIL1/piR-DQ593109 can reduce the blood drug concentration in glioma by reducing the permeability of blood-tumor barrier, thus playing a role in chemotherapy resistance. piR-39,980, PIWIL2 and piR-1037 attenuated the efficacy of chemotherapy by reducing the sensitivity of tumor cells to chemotherapeutic drugs. piR-39,980 and piR-hsa-211,106 enhanced the chemotherapeutic effect by increasing the accumulation of chemotherapeutic drugs in tumor cells, thereby achieving anti-proliferation and pro-apoptosis effects

Despite significant advances in tumor treatments, tumor therapy resistance remains a major challenge. Although most tumors regress rapidly and significantly after initial treatment, clinical drug resistance sets in at a later stage of the treatment, leading to treatment failure and, ultimately, patient death (Fig. 5).

Overexpression of PIWIL1 leads to resistance of MM cells after treatment with chemotherapeutic drugs, whereas its downregulation leads to re-sensitization of MM cells to chemotherapy. Mechanistically, PIWIL1 overexpression reduced the phosphorylation of autophagy-related proteins (mTOR and AKT-Ser473) and increased the expression of PINK1/Parkin pathway protein Parkin, mitophagy-related protein (optineurin) and p-TBK-1, promoting MM cell drug resistance by regulating mitochondrial anti-apoptosis [26]. MM drug resistance is also caused by the blood-tumor barrier (BTB) that restricts the effective delivery of anti-glioma drugs to the affected brain tissues. The knockdown of PIWILI and piR-DQ593109 increased the permeability of BTB by reducing the expression level of tight junction-associated proteins, such as ZO-1, Occludin, and Claudin-5 [255]. High level of piR-39,980 reduced the sensitivity of NB cells to doxorubicin. It increased NB deterioration under doxorubicin treatment by inducing cell growth and enhancing metastasis by targeting JAK3 [343]. The low expression of piR-39,980 leads to increased resistance to chemotherapeutic drugs in DOX-resistance fibrosarcoma cell lines. In terms of mechanism, piR-3998 reduced the resistance of fibrosarcoma cells to DOX and increased DOX sensitivity and intracellular concentrations by targeting and down-regulating RRM2 and CYP1A2. Overexpression of piR-39,980 suppressed CYP1A2, promoting DOX accumulation and increased sensitivity [119]. piR-hsa-211,106 overexpression LUAD cells are sensitive, while piR-has-211,106 knockdown increases LUAD resistance to PDD. piR-hsa 211,106 showed synergistic pro-apoptotic effects with PDD in vitro and in vivo [116]. High levels of piR-1037 promote the resistance of OSCC to CDDP chemotherapy. piR-1037 inhibits CDDP-induced apoptosis by inhibiting Caspase-8 and Caspase-1-dependent death signaling pathways [114]. In HPV-infected cervical epithelium, the oncoproteins E6 and E7 activate PIWIL2, which then reprogram the cells that initiate tumor-initiating cells (TICs) and tumorigenesis of the cervix. In cervical neoplasia, PIWIL2 activates Wnt3a signaling to enhance reprogramming through CBP/ β-catenin interactions and further maintain the stemness of TICs. In addition, blocking the CBP/β-catenin interaction can induce early cell differentiation and increase the sensitivity of cisplatin to a certain extent [317].

#### The name and annotation of piRNAs: database exploration and correction

piRNA is one of three major classes of small regulatory RNAs that are encoded in discrete genomic clusters, many of which are present in the genomic regions of humans [32]. Surprisingly, previous studies have found that a significant number of sequences in different human piRNA databases show a high degree of identity with other ncRNAs, which means that the actual expression of piRNAs may be greatly overestimated [397]. Therefore, constructing a more accurate database plays an extremely important role in the study of piRNAs. In fact, the database has been changed and corrected for generations, and a large number of the above fake piRNAs have been excluded, making the results of the database more realistic. The most commonly used database is piRBase release v3.0. (http://bigdata.ibp.ac.cn/piRBase) [398]. In addition, this database further enriched piRNA targets and realized the visualization of the regulatory network of the targets, which made it more convenient for researchers to explore piRNA functions [399].

## Conclusion and perspective

To date, multiple piRNAs are being continuously discovered and can be accurately identified as abnormal expressions in human diseases. Generally, piRNA and PIWI proteins maintain physiological balance through germ- and somatic cell-mediated synthesis and degradation. However, the disease conditions may occur when the expression of piRNA or PIWI protein is disturbed. We elaborate on recent studies of the abnormal regulatory mechanisms of piRNA/PIWI proteins in various diseases. The preliminary stage of the current research on piRNA/PIWI protein fails to cover its great clinical prospect. Recently, some studies have shown that many piRNAs are highly differentially expressed in fluid, serum, and tissue samples. The specificity and sensitivity of some piRNAs have exceeded that of currently commonly used clinical tumor markers, such as piR-020619 and piR-020450, which are highly expressed in CRC serum, and their sensitivity and specificity have been proved to be better than those of traditional biomarkers CEA and CA-199.

This observation crucially underlines the significant potential of piRNAs as alternatives to current tumor biomarkers. In addition, piRNA/PIWI protein also enhances chemotherapy tolerance by regulating the drug resistance of tumor cells, significantly contributing toward solving the problem of the current widespread clinical chemotherapy resistance and further improving the prognosis of patients. Although the current research on piRNA and PIWI proteins is in the preliminary stage, this cannot mask their great potential in future clinical applications. With the continuous progress of research, we believe that more piRNAs/PIWI protein complexes will be discovered and studied, and eventually be used as specific targets for clinical treatment.

## Data Availability

Not applicable.

## Abbreviations

piRNAs: Piwi-interacting RNAs
RISC: RNA-induced silencing complex
Pol II: RNA polymerase II
H3K9: Histone H3 Lys9
HP1: Heterochromatin protein 1
RDC: Rhino-Deadlock-Cutoff
Trf2: TATA box-binding protein-associated Factor 2
flam: Flamenco
Aub: Aubergine
Ago3: Argonaute 3
zuc: Zucchini
Armi: Armitage
TE: Transposable element
CpG: Cytosine phospho-guanine
DNMTs: DNA methyltransferases
MDSCs: Myeloid-derived suppressor cells
MMSCs: Multiple myeloma stem cells
TSGs: Tumor-suppressor genes
APC: Adenomatous polyposis coli
PTEN: Phosphatase and tensin homolog
GSTP1: Glutathione S-transferase pi 1
tGCI: Transient global cerebral ischemia
HPC: Hypoxic postconditioning
WTAP: Wilms’ tumor 1-associating protein
DLBCL: Diffuse large B-cell lymphoma
FTO: Fat mass and obesity-associated protein
CHAPIR: Cardiac-hypertrophy-associated piRNA
HSF1: Heat shock factor 1
MM: Multiple myeloma
S1PR: Sphingosine-1-phosphate receptor
IKK: IκB kinase
ESCC: Esophageal cell squamous carcinoma
CCL3: Chemokine (C-C motif) ligand 3
ccRCC: Clear cell renal cell carcinoma
PCa: Prostate carcinoma
MMP: Matrix metalloproteinases
PDAC: Pancreatic ductal adenocarcinoma
EMT: Epithelial-mesenchymal transition
TME: Tumor microenvironment
EVs: Extracellular vesicles
DOX: Doxorubicin
RR: Ribonucleotide reductase
ROS: Reactive oxygen species
PC: Pyruvate carboxylase
GSH: Glutathione
MMF: Mycophenolate mofetil
LTR: Long terminal repeat
FAO: Fatty acid β-oxidation
TAM: Tumor-associated macrophages
CDDP: Cisplatin
GC: Gastric cancer
CRC: Colorectal cancer
HIN: High-grade intraepithelial neoplasia
PSCs: pancreatic stellate cells
ICC: Intrahepatic cholangiocarcinoma
HCC: Hepatocellular carcinoma
NSCLC: non-small cell lung cancer
LUAD: Lung adenocarcinoma
LAC: lung adenocarcinomas
BLCA: Bladder cancer
OCa: Ovarian cancer
EOCa: Epithelial ovarian cancer
ENOCa: Endometrioid ovarian cancer
EC: Endometrial cancer
CSCs: Cancer stem cells
cHL: Classical Hodgkin lymphoma
GBM: Glioblastoma
BBB: Blood-brain barrier
BTB: Blood-tumor barrier
OS: Osteosarcoma
AD: Alzheimer’s disease
CYCS: Cytochrome C
ALS: Amyotrophic lateral sclerosis
SALS: Sporadic Amyotrophic Lateral Sclerosis
PASMCs: Pulmonary artery smooth muscle cells
PH: Pulmonary hypertension
RILF: Radiation-induced pulmonary fibrosis
tGCI: Transient global cerebral ischemia
HPC: Hypoxic postconditioning
TICs: Tumor-initiating cells
